# Genome-wide identification and expression analysis of the *GRAS* family under low-temperature stress in bananas

**DOI:** 10.3389/fpls.2023.1216070

**Published:** 2023-08-30

**Authors:** Ning Tong, Dan Li, Shuting Zhang, Mengjie Tang, Yukun Chen, Zihao Zhang, Yuji Huang, Yuling Lin, Zhenguang Cheng, Zhongxiong Lai

**Affiliations:** Institute of Horticultural Biotechnology, Fujian Agriculture and Forestry University, Fuzhou, China

**Keywords:** *Musa acuminata*, *GRAS* family, evolutionary analysis, miRNA, expression pattern

## Abstract

**Introduction:**

GRAS, named after GAI, RGA, and SCR, is a class of plant-specific transcription factors family that plays a crucial role in growth and development, signal transduction, and various stress responses.

**Methods:**

To understand the biological functions of the banana *GRAS* gene family, a genome-wide identification and bioinformatics analysis of the banana *GRAS* gene family was performed based on information from the *M. acuminata*, *M. balbisiana*, and *M. itinerans* genomic databases.

**Result:**

In the present study, we identified 73 *MaGRAS*, 59 *MbGRAS*, and 58 *MiGRAS* genes in bananas at the whole-genome scale, and 56 homologous genes were identified in the three banana genomes. Banana GRASs can be classified into 10 subfamilies, and their gene structures revealed that most banana GRAS gDNAs lack introns. The promoter sequences of *GRASs* had a large number of *cis*-acting elements related to plant growth and development, phytohormone, and adversity stress responsiveness. The expression pattern of seven key members of *MaGRAS* response to low-temperature stress and different tissues was also examined by quantitative reverse transcription polymerase chain reaction (qRT-PCR). The microRNAs-*MaGRASs* target prediction showed perfect complementarity of seven GRAS genes with the five mac-miRNAs. The expression of all seven genes was lowest in roots, and the expression of five genes was highest in leaves during low-temperature stress. The expression of *MaSCL27-2*, *MaSCL27-3*, and *MaSCL6-1* was significantly lower under low-temperature stress compared to the control, except for *MaSCL*27-2, which was slightly higher than the 28°C control at 4 h. The expression of *MaSCL27-2*, *MaSCL27-3*, and *MaSCL6-1* dropped to the lowest levels at 24 h, 12 h, and 4 h, respectively. The *MaSCL*27-4 and *MaSCL*6-2 expression was intermittently upregulated, rising to the highest expression at 24h, while the expression of *MaSCL*22 was less variable, remaining at the control level with small changes.

**Discussion:**

In summary, it is tentatively hypothesized that the *GRAS* family has an important function in low-temperature stress in bananas. This study provides a theoretical basis for further analyzing the function of the banana GRAS gene and the resistance of bananas to cold temperatures.

## 1 Introduction

GRAS [GAI (GIBBERELLIC ACID INSENSITIVE), RGA (REPRESSOR of GAI), and SCR (SCARECROW)] are a family of plant-specific transcriptional regulators with numerous members and widely distributed families. GRAS is involved in almost the whole process of plant growth and development and plays a key role in root and axillary bud development, meristem formation, male gamete development, and gibberellin and light signal transduction (Fode et al., 2008; Torres-Galea et al., 2013). There are some differences in the mode of action and function among different members of GRAS (Morohashi et al., 2003). The proteins of GRAS family members generally vary in size, with canonical GRAS proteins containing 350 to 770 amino acid residues, mostly containing five canonical motifs: LHR I, LHR, VHIID, PFYRE, and SAW. The GRAS family is usually divided into 8-13 subfamilies, with some differences in evolutionary systems between species (Liu and Widmer, 2014). The C-terminal end of the amino acid sequence among members is highly conserved, while the N-terminal end differs greatly, which determines the functional specificity and diversity of the members of the GRAS family (Bolle, 2004).

The identification of the *GRAS* gene family has been reported in several plants species including *Arabidopsis thaliana* (33), rice (60) (Tian et al., 2004), poplar (106) (Liu and Widmer, 2014), water lily (38) (Wang et al., 2016), tomato (53) (Huang et al., 2015), pepper (54) (Zhang et al., 2017; Liu et al., 2018), mung bean (58) (Zhang et al., 2022), barley (62) (To et al., 2020), soybean (117) (Wang et al., 2020), and tea (52) (Wang et al., 2018). The GRAS family classification of different species is slightly different; initially, GRAS was divided into 7 subfamilies, namely, DELLA, SCR, Ls, HAM, PAT1, SHR, and SCL9 (Bolle, 2004), and then it was divided into 8 subfamilies in *Arabidopsis* (a new subgroup SCL3, SCL9 is named LISCL) (Tian et al., 2004). Further, *GRAS* genes were grouped into 14 subgroups in rice and 8-16 subgroups in poplar (Liu and Widmer, 2014), mung bean (Zhang et al., 2022), barley (To et al., 2020), soybean (Wang et al., 2020), and other species.

Accumulated reports have demonstrated that GRASs are vital for plant growth and stress resistance, and different members also have varied functions. The N-termini of different subgroups of the *GRAS* family vary, which may lead to different functions of different subgroups. It is known that PAT is involved in the signal transductions of phytochromes; for example, AtPAT1 and AtSCL21 play a positive regulatory role in the signal transductions of phytochromes A in *Arabidopsis* (Torres-Galea et al., 2013). AtSCL13 acts as a positive regulator of red light signaling and plays a major role in de-yellowing *Arabidopsis* hypocotyl elongation (Torres-Galea et al., 2006). DELLA proteins have important roles throughout intrinsic and extrinsic adversity stress signaling, and they are involved in the CBF protein-induced chilling resistance mechanism to improve plant chilling tolerance (Achard et al., 2008). In addition, it is also involved in gibberellin signaling (Liu and Widmer, 2014) and regulates jasmonic acid signaling and light signaling when interacting with the JAZ1 and PIF (De Lucas et al., 2008; Hou et al., 2010). SHR acts upstream of SCR, and the two interact to regulate root radial signaling pathways (Helariutta et al., 2000), while SCL3 acts downstream of SCR and SHR to regulate the elongation of root cells (Zhang et al., 2011). HAM is involved in stem cell differentiation and proliferation (Geng and Zhou, 2021). NSP1 and NSP2 are involved in the signal pathway of leguminous root nodules (Hirsch et al., 2009). *SCL7* gene responds to low temperature and other stresses in *Populus euonymus (* Ma et al., 2011), *SlGRAS4* responds to low-temperature stress in tomato (Huang et al., 2015), and *PtrGRAS11, PtrGRAS26*, and *PtrGRAS30* respond to low-temperature stress in citrus (Yang et al., 2022).

Several studies have indicated that microR171 can regulate plant growth and development by targeting some members of the *HAM* subgroup of the *GRAS* family. It has been shown that miR171 targets *AtHAM1, AtHAM2*, and *AtHAM3* in *Arabidopsis* to control the proliferation of bud meristem and the formation of axillary buds, and that overexpression of miR171 leads to a reduction in branching in *Arabidopsis (* Schulze et al., 2010; Wang et al., 2010). MiR171 regulates axillary meristem development and flowering time in barley, and overexpression of miR171 can reduce the number of branches and delay flowering (Curaba et al., 2013). In rice, miR171 regulates the transition from vegetative growth to reproductive growth by controlling interstem meristems and flowering by *OsHAM* transcription factor (Fan et al., 2015). In addition, most of the 12 miR171 members are involved in cold stress response (Lv et al., 2010). The expression of miR171d and miR171e was upregulated after cold stress in maize, and it mediated cold stress by participating in the GA signal transduction pathway. In *Arabidopsis*, miR171 was found to be significantly responsive to high salt, drought, and cold stress (Liu et al., 2008), and in wheat, miR171a was found to be significantly downregulated after cold stress treatment (Song et al., 2017). MiR171-*SCL6* in lily regulates somitogenesis and development, especially the development of torpedo-shaped embryos (Li H. et al., 2017).

The banana (*Musa* spp.) is one of the most popular and economically important fruits in the world. Cultivated banana is generally susceptible to external and unstable environmental changes such as exposure to cold stress. The minimum temperature for banana growth and development is 13 °C. When the temperature drops to 5 °C, the leaves begin to suffer from cold damage, at 2.5 °C the leaves are severely damaged, and at 0 °C the plants freeze to death. The lower the temperature or the longer it lasts, the more severely the plants suffer. To further investigate the banana *GRAS* family and improve the resistance of bananas to cold, this study was conducted to identify the *GRAS* transcription factor family in the *Musa acuminata*, *M. balbisiana*, and *M. itinerans* genomic databases based on previous studies on the *GRAS* family. A total of 73 *MaGRAS*, 59 *MbGRAS*, and 58 *MiGRAS* genes were identified. The functional diversity of the banana *GRAS* family was inferred based on *cis*-acting elements, and the presence of low-temperature response elements in most of the banana *GRAS* genes was found, which was hypothesized to have important functions in banana low-temperature stress. Collinearity analysis indicated that tandem and segmental duplications (SD) might be the main reason for the expansion of the banana *GRAS* family.

## 2 Materials and methods

### 2.1 Plant materials and cold treatment

In the present study, uniform and well-growing ‘Tianbaojiao’ banana plantlets with six- to seven-leaf stage were exposed to 4 °C cold treatment in a growth chamber (GXZ-280C, Ningbo Jiangnan Instrument Factory, Ningbo, China) at 28 °C, and to a photoperiod of 16/8 h (day/night, 1500 ± 200 lx). After cold treatment (4 °C), the first fully expanded top leaves were harvested at time points 0, 4, 8, 12, 18, and 24 h. The first leaf (expanded leaf) and different tissue parts (leaf, pseudostem, and root) of the control plants at 28 °C were snap frozen in liquid nitrogen and stored in an ultra-low temperature refrigerator at −80 °C and used for total RNA extraction, qRT-PCR expression analysis, and RLM-RACE test materials.

### 2.2 Identification of *GRAS* gene family members in bananas and phylogenetic tree construction

The genomic and protein data of *M. acuminata* var. *DH-Pahang (* Martin et al., 2016), *M. balbisiana* var. *DH PKW (* Wang et al., 2019), and *M. itinerans* were downloaded from the Banana Genome Database (http://banana-genome-hub.southgreen.fr). Hidden Markov model files of the GRAS structural domains were downloaded from the Pfam (Mistry et al., 2020) database (http://pfam.xfam.org/), and these three genomic data of bananas were searched (E-value ≤ 1e-10) using the HmmSearch program in the Hmmer 3.0 software to obtain preliminary candidate proteins after redundancy removal. The structural domains of the banana MaGRAS, MbGRAS, and MiGRAS candidate proteins were detected by NCBI-CDD (Lu et al., 2019) (https://www.ncbi.nlm.nih.gov/Structure/cdd/cdd.shtml) tool and SMART (Letunic et al., 2020) (http://smart.embl-heidelberg.de/), and the sequences without GRAS structural domains were removed, resulting in 73, 59, and 58 members, respectively. After NCBI-Blastn/Blastp comparison, and with reference to the *Arabidopsis* GRAS nomenclature and the position of the chromosome (chr) where each member is located, each member was named sequentially. Segmental duplications and tandem duplications genes of the *GRAS* family in bananas were analyzed using TBtools (Marques-Bonet et al., 2009). Segmental duplications are usually defined as DNA fragments with highly similar sequences greater than 1 Kb (Chen et al., 2020). Tandem duplications are defined as homologous genes located within 100 kb and separated by 10 or fewer non-homologous spacer genes (Hanada et al., 2008; Zhang et al., 2020). Based on information on the physical location of family members on chr, chr positioning and covariance analysis were mapped using TBtools, and values of non-synonymous substitutions (Ka) and synonymous substitutions (Ks) were calculated for GRAS replicated genes in bananas. The gene duplication occurrence time (T) was calculated as T = Ks/2λ, where the evolutionary rate (λ) of *Musa* was 4.5 × 10^−9^ (Lescot et al., 2008).

A total of 33 *Arabidopsis* and 53 rice GRAS protein sequences were downloaded from the Tair (https://www.arabidopsis.org/) database and transcription factor database (http://planttfdb.cbi.pku.edu.cn/) as comparison sequences. Using the Neighbor-joining method of MEGA 6.06, phylogenetic trees with a total of 265 GRAS amino acid sequences were constructed for three species of banana, *M. acuminata, M. balbisiana*, and *M. itinerans*, *Arabidopsis*, and rice, with the running parameters of Poisson model, complete deletion, and bootstrap value set to 1,000 replicate tests. Other parameter values defaulted.

### 2.3 Analysis of the *GRAS* gene structure and prediction of the structural domains of the proteins

Molecular weight, theoretical isoelectric point, instability coefficient, and signal peptide of banana GRAS protein were predicted using ExPASy’s (Wiederschain, 2006) (https://web.expasy.org/protparam/) and SignaIP 4.1 Server (Petersen et al., 2011) (http://www.cbs.dtu.dk/services/SignalP/). The conserved motifs of banana GRAS protein sequences were analyzed through the MEME (Bailey and Gribskov, 1998) (Multiple Expectation Maximization for Motif Elicitation) online website. The software TBtools was used to map the motifs, phylogenetic trees, gene structures, and protein structural domains on the obtained data.

### 2.4 Diction of *cis*-acting elements of banana *GRAS* promoter and transcription factor binding sites

Sequences 2000 bp upstream of the transcription start site of the *MaGRAS*, *MbGRAS*, and *MiGRAS* genes were used for *cis*-acting element prediction using the PlantCare (Lescot et al., 2002) (http://bioinformatics.psb.ugent.be/webtools/plantcare/html/) online website. Their promoter transcription factor binding sites were predicted using the PlantTFDB (Jin et al., 2016) online website (http://planttfdb.cbi.pku.edu.cn/) with parameters set to p-value ≤ 1e-6. The data obtained was compared, analyzed, and finally plotted by TBtools software.

### 2.5 Regulated miRNA prediction and cleavage site validation of banana *GRAS* family cleavage members

The plant miRNA from the miRbase database and the miRNA mature body sequence identified from the banana transcriptome were used as the database, and the psRNATtarget (Dai and Zhao, 2011) online analysis software (http://plantgrn.noble.org/v1_psRNATarget/) was used for the prediction analysis of miRNAs regulating the *MaGRAS* gene family of banana. The expected value was set to 0–3, and the others were the default parameter values.

Total RNA from the roots, pseudostem, and leaves of ‘Tianbaojiao’ transplants with satisfactory integrity and purity were mixed, and reverse transcribed by RLM-RACE using the GeneRacerTM Kit (Invitrogen, USA) to obtain cleavage site verification cDNA. Cleavage primers of target genes were designed using DNAMAN 6.0 (Lynnon Biosoft), with primers shown in **Table 1**. Two rounds of nested PCR amplification were performed when combined with the universal primers GeneRacer™ 5 ′ Primer, GeneRacer™ 5 ′ Nested Primer, and the specific primers. The primers were synthesized by Fuzhou Shangya Biotechnology Co.

**Table 1:** The primers used for banana miR171 target genes cleavage site verification.

Gene	miRNA	Use	Primer sequences(5′→3′)
*MaSCL27-1*	mac-miR171a-3p.1 mac-miR171a-3p.2	5′R1	GCATGAAGGAGGACCATTG
		5′R2	CGAAGTCGATGATGTGAATACG
*MaSCL27-2*	mac-miR171a-3p.1 mac-miR171a-3p.2	5′R1	TGAGCTTGTGCACAACGTC
		5′R2	AGGTTGGAGTTGCAGAGAGG
*MaSCL27-3*	mac-miR171a-3p.1 mac-miR171a-3p.2	5′R1	CCACCAAACCCGATGTCAGAGT
		5′R2	GATGGGAGAGACCTCAGAGAAC
*MaSCL27-4*	mac-miR171a.2 mac-miR171b.2 mac-miR171c.1	5′R1	ATGTCCCATTGAGTCGTGAGG
		5′R2	GAGACGAGAACTGACTCTGCC
*MaSCL6-1*	mac-miR171a-3p.1 mac-miR171a-3p.2	5′R1	GTGAAGCTGGCGAATTGGAC
		5′R2	GTCGGAGAAGGTCTTGTAAGCTC
*MaSCL6-2*	mac-miR171a-3p.1 mac-miR171a-3p.2	5′R1	TGGATGGAGGTGAAGATGG
		5′R2	CCAAGCTTGAGCACAACGT
*MaSCL22*	mac-miR171a-3p.1 mac-miR171a-3p.2	5′R1	GTGAAGCTTGTGAATTGGACGATG
		5′R2	ATGGTGTGGAGATAGCGGAT

The PCR reaction system was 25 µL, Dream TaqTM Green PCR Master Mix (2X) 12.5 μL, ddH2O 9.5 μL, template cDNA 1 µL, and 1 µL each of the upstream and downstream primers. The amplification procedure was 94 °C predenaturation for 3 min, 94 °C denaturation for 30s, 55 to 60 °C annealing for 30s, 72 °C extension for 30s, 34 cycles, and the last 72 °C extension for 10 min. The amplified products were detected by 1% agarose electrophoresis, and the target bands were recovered by Gel/PCR Extraction Kit. After being ligated into the pMD 18-T vector and transformed into DH5α sensors for bacteriophage PCR, several single colonies were picked for sequencing after TA cloning. The sequencing of the samples was entrusted to Shanghai Boshang Biotechnology Co.

### 2.6 The qPCR analysis of banana miR171 and its target genes

According to the results predicted by psRNATarget prediction, the key members of the *MaGRAS* gene family were further selected, and qPCR-specific primers were designed on both sides of the cleavage site. The key members of the *MaGRAS* gene family were analyzed for their specific expression in different tissue sites of bananas and under low-temperature stress. Primer design was performed using DNAMAN 6.0 with a target fragment size of 80–160 bp, and the primer sequence and annealing temperature are shown in **Table 2**.

**Table 2:** Oligonucleotide primers for real-time PCR analysis of *MaGRAS* genes.

Gene	Primer sequences(5′→3′)	Size(bp)	Tm (°C)
*MaSCL27-1*	qF: TGCAAAGATGGTCGAGGC qR: TGTCAGAGTGGAAGGGAGGA	140	60
*MaSCL27-2*	qF: GCACTTACAACACGCATTGG qR: AAAGCAGAGCGGATAAGGG	114	60
*MaSCL27-3*	qF: GTAATTTCTCTGGCGCGC qR: GTTCGAGAGGATGAGTTGCA	87	59
*MaSCL27-4*	qF: AGCAGCAGCATCATCATCAG qR: AAAGCAGAGCGGATAAGGG	124	60
*MaSCL6-1*	qF: GAATTGGTTGACCAGCTCTTC qR: GGGCCTCCTTGAAGTAGAAG	110	58
*MaSCL6-2*	qF: AGTTGATCGAGGTCGGGAA qR: CCAACGGCGTCAAGATTC	152	60
*MaSCL22*	qF: GAAGCAGGGAACATCGTCAG qR: TGTGGAGATAGCGGATGAAGA	133	60
*CAC*	qF: AACTCCTATGTTGCTCGCTTATG qR: GGCTACTACTTCGGTTCTTTCAC	148	57
mac-miR171a-3p.1	TGATTGAGCCGCGCCAATATC		66
mac-miR171a-3p.2	TGATTGAGCCGCGCCAATAT		66
mac-miR171a.2	TGATTGAGCCGTGCCAATAT		63
mac-miR171b.2	TGATTGAGCCGTGCCAATATC		63
mac-miR171c.1	GTGATTGAGCCGTGCCAATATT		63
*U6*	CATCCGATAAAATTGGAACGA		63

We took samples of roots, false stems, and leaves of transplanting seedlings under 28 °C control (CK) and 4 °C cold treatment for 4 h, 8 h, 12 h, 18 h, and 24 h, and then extracted the total RNA of each sample according to the operating instructions of RNAprep Pure Plant Kit (TIANGEN, China). The concentration, purity, and integrity of the extracted RNA were determined by 1.0% non-denaturing agarose gel electrophoresis and a UV spectrophotometer. RNA processed at different tissue and various time points was reverse-transcribed to cDNA as a template for qPCR using PrimerScriptTM RT Reagent Kit (Perfect Real Time) reverse transcription kit (Takara, Japan), according to the manufacturer’s instructions. *CAC* was used as the internal reference gene (Chen et al., 2011), and *U6* was used as the internal reference gene for miRNA. Roche LightCycler 480 fluorescence quantitative PCR instrument was used to amplify the miRNA. Three biological replicates were performed for each sample, and the mean value was taken. The relative expression level of the target gene was calculated by 2^−ΔΔCT^, and Microsoft Excel 2016 and GraphPad Prism version 8.0.2 for Windows (GraphPad Software, San Diego, California USA, www.graphpad.com) were used for calculation analysis and mapping. IBM SPSS Statistics for Windows, version 26.0 (IBMCorp., Armonk, N.Y., USA) was used for significance analysis of the expression level of each gene member.

## 3 Results and analysis

### 3.1 Identification of GRAS family members and basic physical and chemical characteristics of proteins in bananas

NCBI-CDD and SMART yielded 73 MaGRAS, 59 MbGRAS, and 58 MiGRAS family members. Coding sequences (CDS) alignment analysis using NCBI-BLAST revealed that some *MaGRAS* genes contained multiple transcripts; *Ma03_g02280* and *Ma08_g09130* had three transcripts each, and *Ma07_g05950* and *Ma11_g18690* had two transcripts each. The resulting members were named sequentially according to the genome database annotation, coding sequences chromosomal mapping information, and the NCBI-Blastp/Blastn alignment results, and with reference to the *Arabidopsis* GRAS nomenclature method.

ExPASy’s online tool ProtParam was used to analyze the basic physicochemical properties of the proteins encoded by the banana GRAS family (**Supplementary Table 1**). The number of amino acids in each member of the banana MaGRAS family ranged between 401 and 805 amino acids (aa), and protein molecular weight was between 44902.63–90217.59Da. The theoretical isoelectric points were located between 4.79 and 8.92, and the instability coefficient was between 42.12–565. The number of amino acids for each member of the MbGRAS family was between 316–991 aa, and the protein molecular weight was 33149.59–108846.87Da. The theoretical isoelectric points were found between 4.43 and 8.87, and the instability coefficient was between 40.77–66.99. The number of amino acids in each member of the MiGRAS family was between 338–888 aa, and protein molecular weight was between 37943.74–98811.03Da. The theoretical isoelectric points were between 4.85 and 9.14, and the coefficient of instability was between 42.03–67.58. The theoretical average values of other electric points of MaGRAS, MbGRAS, and MiGRAS were 6.04, 5.91, and 5.91, respectively, and the instability coefficient was greater than 40, indicating that most of them were weakly acidic and unstable proteins.

### 3.2 Chromosome localization and collinearity analysis of banana *GRAS*

**Figure 1:**
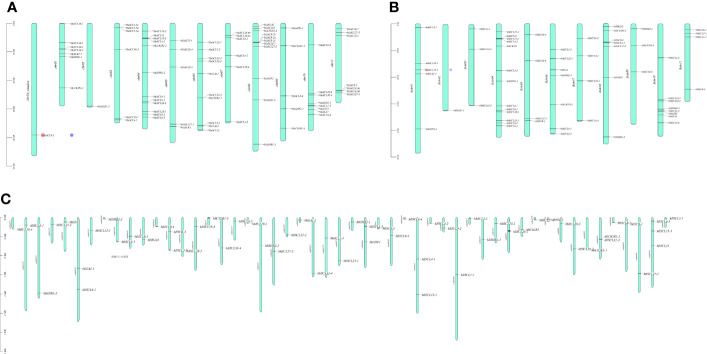
Chromosomal localization of *GRAS* genes in bananas. **(A–C)** are the chromosomal localization of the *MaGRAS*, *MbGRAS*, and *MiGRAS* genes of bananas, respectively.

Chromosomal localization results of the *GRAS* gene family in bananas showed that the 73 *MaGRAS* members were unequally distributed on 12 chrs, with chrUn_random and chr 2 having the least distribution, both having only one member. Chr 4 had the most distribution with 12 members, followed by chr 6, 8, and 10 with 10, 9, and 8 members, respectively (**Figure 1A**). A total of 59 *MbGRAS* members were distributed on 11 chr. Chr 2 had the least distribution with only one member, chr 4 had the most distribution with 13 members, followed by chr 6, 8, and 10 with 7, 7, and 8 members, respectively (**Figure 1B**). *M. itinerans* assembled only on the scaffold level, whereas *MiGRAS* was localized on 51 scaffolds(S). All had only one member except for S333, S658, S1345, S1584, and S3004, which had two members each, and S784, which had three members (**Figure 1C**).

**Figure 2:**
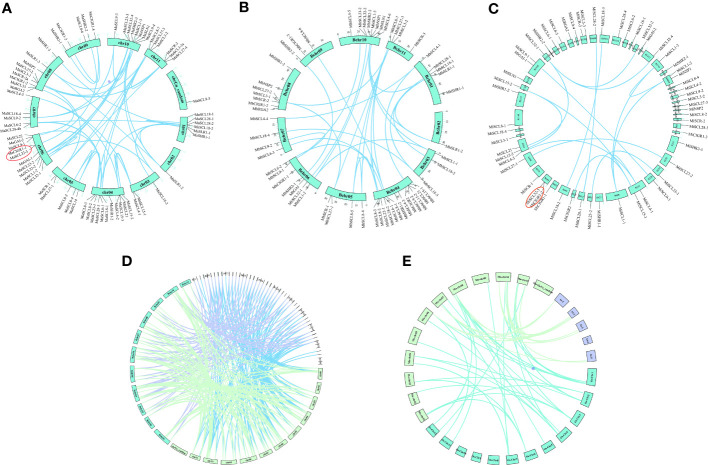
**(A–C)** are the co-linear distributions of banana *MaGRAS*, *MbGRAS*, and *MiGRAS* genes, respectively. **(D)** is the co-linear distribution of banana *MaGRAS, MbGRAS*, and *MiGRAS* genes. **(E)** is the co-linear distribution of banana, rice, and *Arabidopsis GRAS* genes. The line indicates the fragment duplication gene pairs of *GRAS*, and the red circles indicate the tandem duplication pairs.

To investigate the gene duplication events in the *GRAS* family of bananas, co-linear analysis was performed on the *GRAS* genes of banana, rice, and mimosa (**Figures 2A–E**). There were 43 segmental duplication pairs and 1 tandem duplication pair *(Ma06_g25650 and Ma06_g25670*) in *MaGRAS*, distributed on all chrs except chrUn_random. There were 28 segmental duplication gene pairs in *MbGRAS* with no tandem duplication, and at least 2 segmental duplication genes were present on each chr. There were 16 segmental duplication gene pairs and 1 tandem duplication gene pair (*Mi_g027597* and *Mi_g027598*) in *MiGRAS*, unevenly distributed over 20 scaffolds. *GRAS* covariance analysis of the three genomes of bananas revealed 121 homologous gene pairs between *MaGRAS* and *MbGRAS*, 95 homologous gene pairs between *MaGRAS* and *MiGRAS*, and 70 homologous gene pairs between *MbGRAS* and *MiGRAS*. In addition, many *GRAS* genes were found to be relatively conserved among bananas, and 56 of the 73 *MaGRAS* genes were homologous in both *MbGRAS* and *MiGRAS*. Moreover, banana *MaGRAS* was also homologous in rice and mimosa, with co-linearity between 12 *AtGRAS* and 9 *MaGRAS* and between 19 *OsGRAS* and 24 *MaGRAS.*

The Ka and Ks values of the orthologous gene pairs of the banana *GRAS* family were calculated and analyzed whether the family was under natural selection pressure and traced its duplication time during evolution (**Supplementary Table 2**). The Ka/Ks values of all fragment repetitive gene pairs within the banana *GRAS* family were less than one, indicating that the banana *GRAS* genes were subject to purifying selection during evolution. Prediction of the time of *GRAS* gene duplication events in bananas showed that the time of duplication events in the *MaGRAS*, *MbGRAS*, and *MiGRAS* families was 39.3333–93.4333 million years ago, 41.6936–268.5561 million years ago, and 50.3393–360.9675 million years ago, respectively. It suggests that the *MiGRAS* gene may be the earliest to start the duplication event, followed by the *MbGRAS* gene and, finally, the *MaGRAS* gene.

### 3.3 Gene structure and protein domain analysis of GRAS in bananas

**Figure 3:**
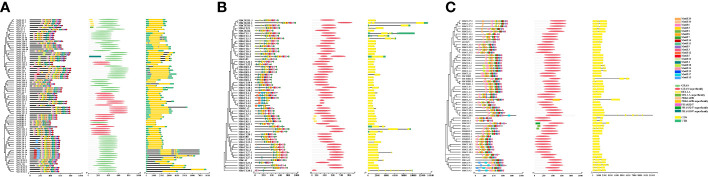
The analysis of gene structures and protein domains of *GRAS* genes. **(A–C)** are the gene structures and protein domains of banana *MaGRAS*, *MbGRAS*, and *MiGRAS* genes, respectively.

Analysis of the exon–intron structure of the banana *GRAS* gene family revealed that *MaGRAS* has 1–3 exons and 0–4 introns (**Figure 3**). Six members contain three exons, nine members contain two exons, and all other members have only one exon. Most members do not contain introns, two members contain four introns, and three members have three introns. *MbGRAS* has 1–11 exons and 0–10 introns, only one member contains 11 exons and 10 introns. Most members have only one exon and no introns, and about a quarter of the members contain 2–4 exons and introns. *MiGRAS* has 1–9 exons and 0–8 introns, about half of the members have only one exon and no introns, and about one-sixth of the members have more than 3 exons or more than 2 introns.

Predictive analysis of the functional structural domains of banana GRAS proteins using TBtools software showed that the conserved structural domains of GRAS were incomplete for four members (MbSCL4-3, MbSCL4-5, MbSCL4-6, and MbSCL28-2). Whereas the remaining members had a complete GRAS superfamily or GRAS conserved domains. In addition to the common GRAS domain, some members also contained other domains. MaSCR-2a had a PHA03307 superfamily functional domain, MiSCL3-3 had a PRK14698 superfamily and a PHA03247 superfamily functional domain, whereas MaSLR1-2, MaSLR1-3, MaSLN1, MaSLR1-1, MbSLN1, MbSLR1-2, MbSLR1-1, MiGAI-1, and MiSLN1 all had a DELLA functional domain. That might be related to their diversity of functions.

The 20 conserved motifs of the banana GRAS proteins were analyzed using the MEME online website and it was found that each MaGRAS contained motif 1, motif 2, motif 4, and motif 6. Except for a few MaGRAS proteins with partial deletions, the rest contained motif 5, motif 7, motif 8, motif 9, and motif 11. Motif 14 and motif 20 were only present in the LISCL subfamily, motif 15 and motif 16 were only present in the AtPAT1 subfamily, and motif 18 was only present in the AtSCR subfamily. Most of the MbGRAS proteins contained motif 1, motif 2, motif 3, motif 4, motif 5, motif 6, motif 7, motif 8, motif 10, motif 11, motif 15, and motif 16. Motif 14 and motif 17 were present only in the AtSCL4/7 subfamily, whereas motif 20 was only present in the AtPAT1 subfamily. Most proteins of the MiGRAS contained the conserved motif, motif 1, and motif 13, besides motif 15 in the LISCL subfamily, motif 16 and motif 20 in the HAM subfamily, and motif 18 in the AtPAT subfamily. The c-terminus sequence of the GRAS proteins was highly conserved and consisted of five motifs in sequence, namely, HLR I, VHIID, LHR II, PFYRE, and SAW 5, present in motifs 1–9, motif 11, and motif 17 of MaGRAS, motifs 1–8, motif 10, and motif 15 of MbGRAS, and motifs 1–7, motif 9, motif 10, and motif 13 of MiGRAS. The majority of motifs of GRAS protein appeared at the C-terminus, suggesting that the family proteins are highly conserved at the C-terminus. The N-terminus motifs, on the other hand, were more discrete, indicating that there may be dissimilarities in the function of the banana GRAS family.

### 3.4 GRAS phylogenetic tree analysis

**Figure 4:**
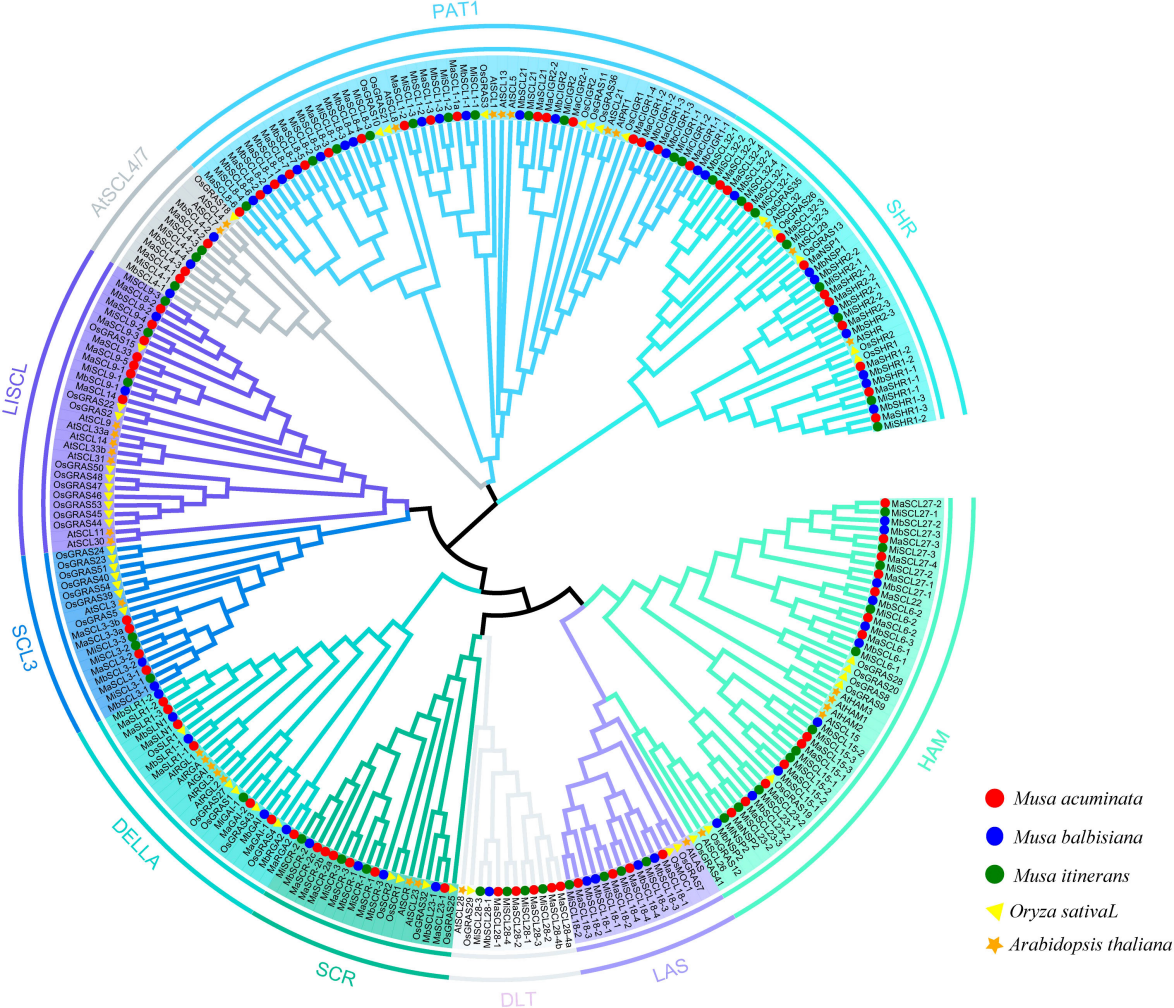
Phylogenetic analyses of GRAS proteins in *M. acuminata*, *M. balbisiana*, *M. itinerans*, rice, and *Arabidopsis*.

To analyze the phylogenetic relationships between GRAS proteins from rice and *Arabidopsis* and GRAS proteins from MaGRAS, MbGRAS, and MiGRAS, 265 GRAS protein sequences from bananas, *Arabidopsis*, and rice were used to construct phylogenetic trees using the Neighbor-joining method of the software MEGA 6.06. The typical GRAS domain minimum length is about 350 amino acids, and some members of the GRAS family in bananas, rice, and *Arabidopsis* have a GRAS domain of less than 350 amino acids and are not reliable, so these members were excluded when constructing the phylogenetic tree (**Figure 4**). The GRAS family of bananas can be divided into 10 subfamilies: SCR, SHR, DELLA, PAT1, HAM, LISCL, LAS, SCL3, SCL4/7, and DLT. The number of members of each subfamily varies, and the most numerous subfamily is PAT1. Each subfamily contains *MaGRAS*, *MbGRAS*, *MiGRAS*, *AtGRAS*, and *OsGRAS* genes. OsGRAS4, OsGRAS19, and OsGRAS43 have previously been reported as rice-specific proteins (Liu and Widmer, 2014), but the results of this evolutionary tree indicate that there are multiple banana GRAS proteins clustered within a subfamily with each of these two rice genes. This suggests that OsGRAS4, OsGRAS19, and OsGRAS43 are homologous to these banana GRAS proteins.

### 3.5 Analysis of promoter *cis*-acting elements and transcription factor binding sites of *GRAS* family members in bananas

#### 3.5.1 Analysis of promoter *cis*-acting elements

**Figure 5:**
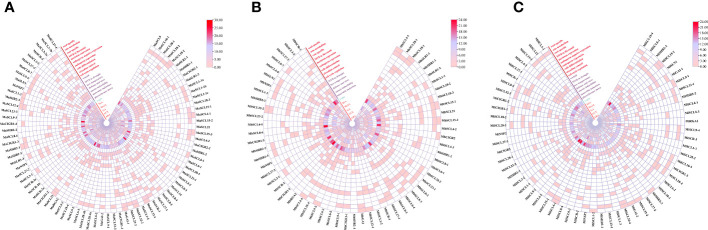
The identified *cis*-acting elements in *GRAS* gene family promoters. **(A, B, C)** are *cis*-acting elements identified in the promoters of *MaGRAS*, *MbGRAS*, and *MiGRAS* genes, respectively.

Predictive analysis of *cis*-acting elements of the banana *GRAS* promoter revealed a large number of CAAT-C A T-box and TATA-box core elements in the promoter region of this gene family. The number of the two core elements contained in different members varied greatly, implying that there are large differences in the transcriptional regulation of banana *GRAS* genes. The banana *GRAS* gene family also has many light response elements, hormone response elements, stress response elements, and plant growth and development-related *cis*-acting elements (except *MiRGA2*, which does not have these *cis*-acting elements). It is speculated that the expression of banana *GRAS* may be regulated by a variety of factors. Different *cis*-elements and their number of loci with the same or similar functions were classified and counted (**Figure 5**).

Banana *GRAS* genes have many photoresponsive elements (except *MiRGA2*), and *MaSCL4-3* and *MaSCL8-3* contain the largest number (25). The banana *GRAS* family contains five hormone-response elements, namely, abscisic acid, auxin, gibberellin, methyl jasmonate, and salicylic acid response elements. All members except *MaSCL1-1a*, *MaSCL1-1b*, and *MaSCL1-1c* contain at least one hormone response element. Most members contain three or more, and 22 members contain five hormone response elements. Further analysis of phytohormone response elements revealed that the largest number of *cis*-elements related to the response of MeJA, with 82% of the members containing at least 6, and some even 18 (*MaSCL4-2* and *MaSLR1-3*). This was followed by the ABA response elements, with 73% of the members containing it. Finally, SA (59%), GA (57%), and AUX (43%) were found. According to the abovementioned analysis, it shows that each member of the banana *GRAS* family gene is involved in different hormone signaling pathways and has a different division of labor in the hormone response.

During their growth and development, plants have complex environmental conditions, and they will face various biological and abiotic stresses, such as low temperature, drought, injury, and pathogens. Multiple stress response elements exist in the promoter region of the banana *GRAS* gene family, namely, anaerobic induction (ARE), hypoxia-specific induction (GC-motif), defense and stress (TC-rich repeats), low temperature (LTR), drought-induced MYB binding point (MBS), and injury response elements (WUN-motif). Besides, multiple *cis*-acting elements are also present in the banana *GRAS* promoter regulatory sequence, both specific and related to tissue growth and development. These include a root-specific element (motif 1), seed-specific regulatory element (RY-element), cell cycle regulatory element (MSA-like), circadian regulatory element (circadian), palisade mesophyll cell differentiation regulatory element (HD-Zip1), endosperm expression element (GCN4_motif), endosperm-specific negative expression regulatory element (AACA_motif), meristem expression (CAT-box) regulatory element, MYB binding point (MBSI) of flavonoids biosynthesis genes, and cornicin metabolism regulatory element. The aforementioned results suggest that the banana *GRAS* family may not only regulate plant growth and development but also respond to stress.

#### 3.5.2 Analysis of transcription factor binding sites

**Figure 6:**
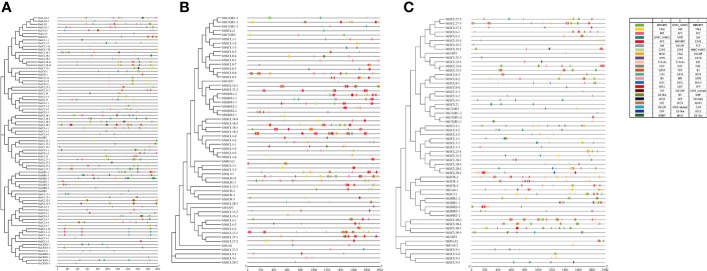
Prediction of transcription factor binding sites in the promoters of *MaGRAS* **(A)**, *MbGRAS* **(B)**, and *MiGRAS* **(C)**. Boxes of different colors represent different transcription factor families.

To explore the interaction of transcription factors and banana *GRAS*, the transcription factor binding site (TFBS) in its promoter region was predicted and analyzed (**Figure 6**). A total of 23 families of transcription factors (TF) families (BBR-BPC, AP2, B3, BES1, bHLH, bZIP, C2H2, C3H, CPP, Dof, ERF, G2-like, GATA, HD-ZIP, HSF, MIKC_MADS, MYB, NAC, TALE, TCP, Trihelix, WOX, and WRKY) were excavated in the *MaGRAS* promoter region. The largest number of BBR-BPC binding sites (1,100) were present in 43 members, followed by MIKC_MADS (419) and AP2 (341), both present in 42 members; the least were C3H, CPP, Trihelix, and WRKY, all with only one binding site. A total of 23 transcription factor families were also present in the promoters of both *MbGRAS* and *MiGRAS genes*, and they both had 20 transcription factor families identical to *MaGRAS*. Trihelix was present only in *MaGRAS* and *MbGRAS*, WRKY was present only in *MaGRAS* and *MiGRAS*, MYB_related was present only in *MbGRAS* and *MiGRAS*, and HSA, E2F/DP, and Nin-like were present only in *MaGRAS*, *MbGRAS*, and *MiGRAS*, respectively. In addition, there were some differences in the type, number, and distribution of transcription factors in the promoters of each member of the banana *GRAS* family, for instance, 11 transcription factor family binding sites in the *MbSLR1-1* promoter. However, no transcription factor was absent in the *MaSCL9-1, MaNSP2, MaCIGR1-4, MbSCR-1, MiSHR2-2*, and *MiNSP2* promoters; and there were 321 transcription factor binding sites in *MbSCL18-2.* However, only one transcription factor-binding site was included in *MaSHR2-2, MaSCL4-2, MaNSP1, MaGAI-1, MbSCL21, MbSCL4-5, MbNSP1, MiSCL8-4, MiNSP1*, and *MiCIGR1-3*. Therefore, it is speculated that the *GRAS* family plays multiple roles in banana growth and development and that different members may play different roles.

### 3.6 Identification of MaGRAS genes targeted by miRNAs and validation of cleavage sites

The miRNA-regulating *MaGRAS* family members were predicted using psRNATarget online prediction software. The analysis showed that when the E value was 0–3, 14 members were shown to be targets of 38 miRNAs. Among them, *MaSCL27-1*, *MaSCL27-2*, *MaSCL27-3*, *MaSCL27-4*, *MaSCL6-1*, *MaSCL6-2*, *MaSCL22*, *MaSCL15-1*, *MaSCL15-2*, and *MaSCL15-3* were shown to be targets of 22 microRNAs of the miR171 family. *MaSCL15-3* was also targeted by five miR408. *MaCIGR2-1* can be targeted by 3 miR172, and *MaNSP2* was shown to be a target of 10 microRNAs of the miR171 family (**Supplementary Table 3**).

Further study found that miR171 acted in a manner similar to siRNA, but somewhat different from other miRNAs, with almost all miRNAs acting in a completely complementary pairing manner. Therefore, when the E value was *0, MaSCL27-1, MaSCL27-2, MaSCL27-3, MaSCL6-1, MaSCL6-2, and Mascl22*, which are regulated by both mac-miR171a-3p.1 and mac-miR171a-3p.2, and *MaSCL27-4*, which is also regulated by mac-miR171a.2, mac-miR171c.1, and mac-miR171b.2, were selected as candidate members for subsequent expression analysis (**Table 3**).

**Table 3:** Prediction of miRNA target genes in the *MaGRAS* family.

MiRNA	Homologous miRNA	Gene	Target site	Inhibition
mac-miR171a-3p.1	ath-miR171a-3p	*MaSCL27-2*	1299-1319	Cleavage
		*MaSCL22*	426-446	Cleavage
		*MaSCL27-1*	1296-1316	Cleavage
		*MaSCL6-2*	1122-1142	Cleavage
		*MaSCL27-3*	1311-1331	Cleavage
		*MaSCL6-1*	1146-1166	Cleavage
mac-miR171a.2	htu-miR171a	*MaSCL27-4*	1315-1334	Cleavage
mac-miR171c.1	mtr-miR171c	*MaSCL27-4*	1314-1334	Cleavage
mac-miR171b.2	osa-miR171b	*MaSCL27-4*	1314-1334	Cleavage
mac-miR171a-3p.2	zma-miR171a-3p	*MaSCL6-2*	1123-1142	Cleavage
		*MaSCL22*	427-446	Cleavage
		*MaSCL27-3*	1312-1331	Cleavage
		*MaSCL6-1*	1147-1166	Cleavage
		*MaSCL27-2*	1300-1319	Cleavage
		*MaSCL27-1*	1297-1316	Cleavage

**Figure 7:**
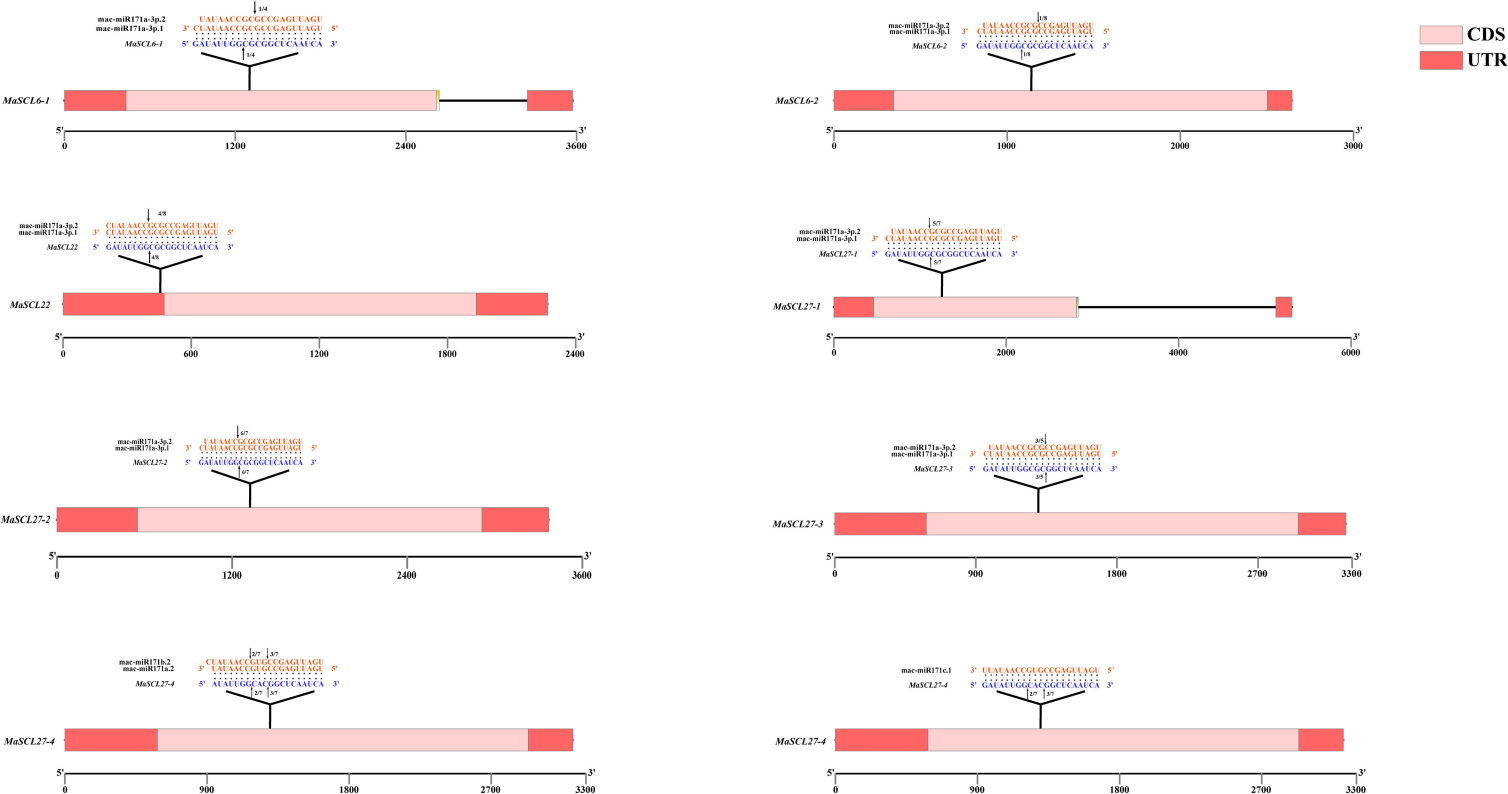
Action site comparison and cleavage site validation of miRNA-targeted regulation of the *MaGRAS* gene. Black arrows indicate the cleavage sites, and the numbers indicate the fraction of cloned PCR products at different positions.

The RLM-RACE method has been used for showing cleavage site verification (**Figure 7**). *MaSCL6-1, MaSCL6-2, MaSCL22, MaSCL27-1, MaSCL27-2*, and *MaSCL27-3* were all cleaved by both mac-miR171a-3p.1 and mac-miR171a-3p.2, all of which produced single cleavage sites. In addition, *MaSCL27-4* was simultaneously cleaved by miR171a.2, miR171b.2, and miR171c.1, generating two cleavage sites. The shear sites of mac-miR171a-3p.1 for *MaSCL6-1, MaSCL6-2, MaSCL22, MaSCL27-1*, and *MaSCL27-2* were located between nucleotides at positions 8 and 9 of the binding region. The *MaSCL27-3* cleavage site was between the nucleotides at positions 11 and 12 of the binding region. The shear sites for *MaSCL22, MaSCL27-1*, and *MaSCL27-2* were between the nucleotides at positions 7 and 8 in the binding region. However, the MaSCL27-3, *MaSCL*6-1, and *MaSCL*6-2 cleavage sites were between the nucleotides at positions 10 and 11 of the binding region. It indicates that the same miRNA can mediate different members of the same target gene family with the same shear site and can be involved in regulating the expression of target genes simultaneously. In addition, *MaSCL27-4* can be cleaved by mac-miR171a.2, mac-miR171b.2, and mac-miR171c.1 simultaneously, producing two cleavage sites at positions 7–8/11–12, 8–9/11–12, and 8–9/11–12 of the binding region, respectively. Interestingly, these miR171 sequences complementarily recognized with target genes can be fully paired and almost all of the shearing sites were located at the GC where the miRNA binds to the target genes.

### 3.7 Expression analysis of some *MaGRAS* members in different tissue parts of ‘Tianbaojiao’

**Figure 8:**
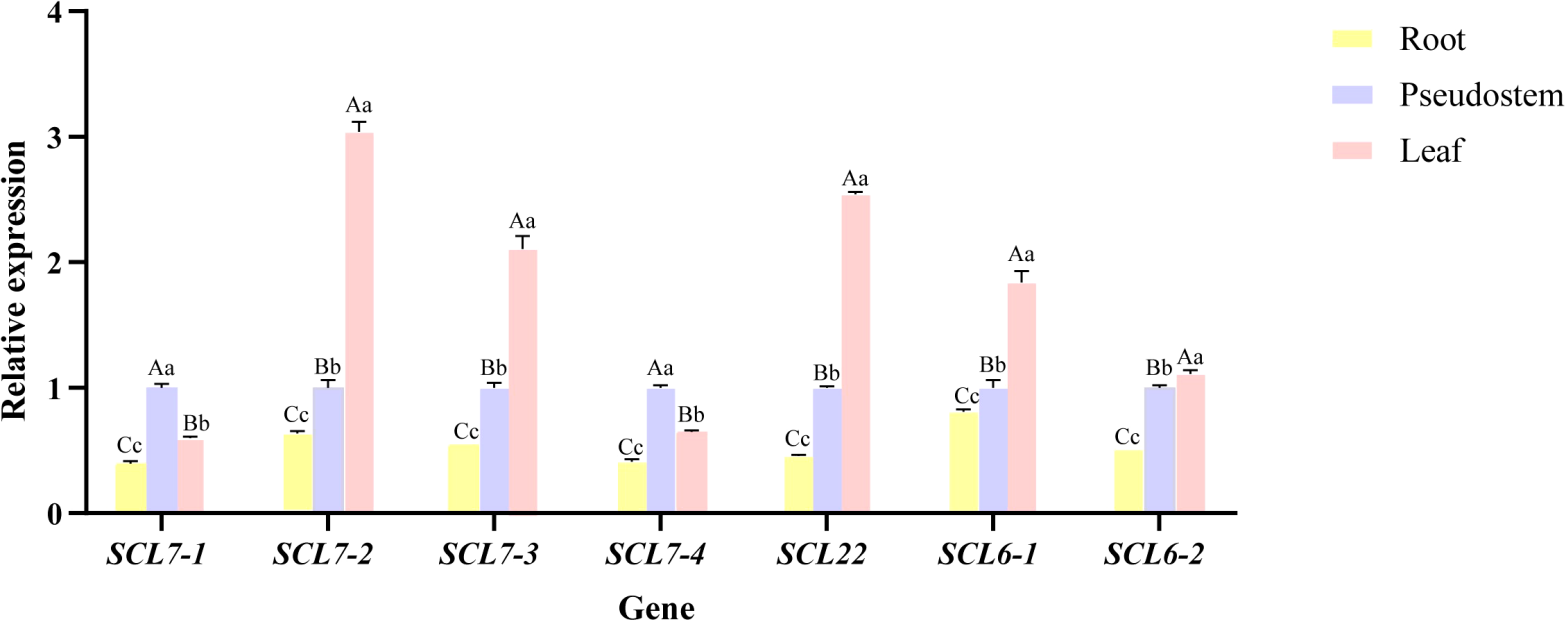
Relative expression levels of *MaGRAS* genes in different tissues. Control (CK) at 28 °C and low-temperature treatment at 4 °C for 4 h, 8 h, 12 h, 18 h, and 24 h. Capital letters indicate significant differences at p<0.01 and lowercase letters indicate significant differences at p<0.05.

The expression of seven key *MaGRAS* members (*MaSCL27-1, MaSCL27-2, MaSCL27-3, MaSCL6-1, MaSCL6-2, MaSCL22*, and *MaSCL27-4*) targeted by miR171 was analyzed in different tissue parts of ‘Tianbaojiao’ transplants using *CAC* as an internal reference gene (**Figure 8**). The seven key members of the *MaGRAS* gene family were expressed in roots, leaves, and pseudostems, but the expression was lower in roots than in leaves and pseudostems. Except for *MaSCL27-1* and *MaSCL27-4*, the expression of the other five genes was significantly higher in leaves than in pseudostems, reaching a highly significant difference (P<0.01), and their expression patterns were more similar in each tissue site. Overall, *MaGRAS* genes showed low levels of expression in both roots and high levels in leaves and pseudostems, with most members reaching the highest expression in leaves.

### 3.8 Expression analysis of *MaGRAS* and mac-miR171 under cold stress

**Figure 9:**
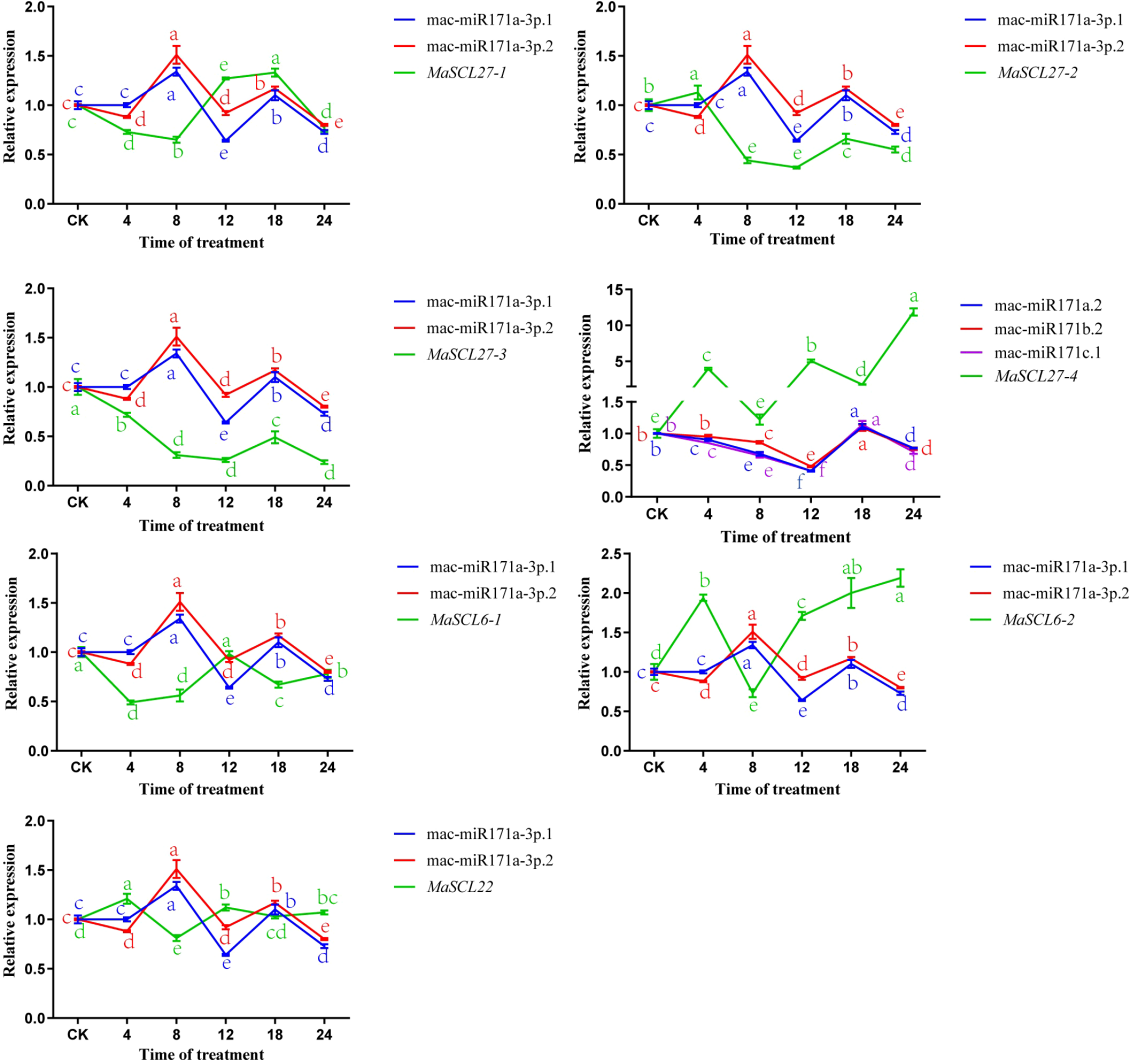
Relative expression levels of *MaGRAS* genes and mac-miR171 under cold stress. Lowercase letters indicate significant differences at p<0.05.

The results of the relative expression analysis of different *MaGRAS* members under low-temperature stress are shown in **Figure 9**. The expressions of all seven *MaGRAS* members were downregulated at 8 h of low-temperature treatment in ‘Tianbaojiao’ transplants compared with the 28 °C control. In addition, the expression patterns of the seven *MaGRAS* during low-temperature stress compared with the 28 °C control level can be divided into two categories. In the first category, *GRAS* expression levels were positively regulated by intermittent upregulation with increasing low-temperature stress. *MaSCL27-4, MaSCL6-2*, and *MaSCL22* were upregulated or significantly upregulated at 4 h, 12 h, and 24 h, and *MaSCL27-1* was significantly upregulated from 12 h to 18 h. The second category was the continuous negative regulation of *GRAS* expression level with the increasing degree of low-temperature stress. The expression of *MaSCL27-3* and *MaSCL6-1* was always lower than that of the control without low-temperature treatment, and the expression was lowest at 24 h and 4 h, respectively, while the expression of *MaSCL27-2* was continuously lower than that of the control after 4 h and dropped to the lowest at 12 h.

The expression patterns of *MaSCL27-1, MaSCL27-2, MaSCL27-3, MaSCL27-4, MaSCL6-1, MaSCL6-2*, and *MaSCL22*, and mac-miR171a-3p.1, mac-miR171a-3p.2, mac-miR171a.2, mac-miR171c.1, and mac-miR171b.2 were analyzed under low-temperature stress at 4 °C, using U6 as an internal reference gene (**Figure 9**). Compared with CK, the expression of mac-miR171a-3p.1 and mac-miR171a-3p.2d significantly increased at 8h and 18h, respectively, and significantly decreased at 12h and 24h, respectively. The expression of mac-miR171a.2, mac-miR171c.1, and mac-miR171b.2 increased significantly at 18h and decreased significantly at 8h, 12h, and 24h, respectively. *MaSCL27-4* was largely negatively correlated with miR171a.2, miR171b.2, miR171c.1, and *MaSCL22* and *MaSCL6-1* were also largely negatively correlated with miR171a-3p.1 and miR171a-3p.2. In addition, miR171a-3p.1 and miR171a-3p.2 inhibited their targets (*MaSCL6-2*, *MaSCL27-1*, *MaSCL27-2*, and *MaSCL27-3*) during the pre-low-temperature stress period. To some extent, miRNAs are confirmed to negatively regulate the expression of target genes at the post-transcriptional level.

## 4 Discussion

### 4.1 The *GRAS* gene family shows expanded and functionally diverse in bananas

The GRAS gene family regulates plant growth and development, including GA signaling, root and axillary shoot development, and response to abiotic stress. They have been identified in a variety of plants, but have been reported relatively rarely, in bananas, especially as genome-wide identifications. Based on genomic data of *M. acuminata*, *M. balbisiana*, and *M. itinerans*, 73 *MaGRAS*, 59 *MbGRAS*, and 58 *MiGRAS* genes, respectively, were identified by bioinformatics methods. Four of the *MaGRAS* genes had more than one transcript (*Ma03_g02280, Ma07_g05950, Ma08_g09130*, and *Ma11_g18690*), with significant amplification of membership compared to various species such as *Arabidopsis* (33), water lily (38), and tomato (53). In addition, the *GRAS* gene number in *M. acuminata* banana was the highest, which may be the largest by this genome. Chromosome localization information showed that *MaGRAS* and *MbGRAS* genes were unevenly distributed on all the banana chrs, and only one *GRAS* gene was found on chr2, while most members were distributed on chr4 and formed short clusters of tandem repeats. Most *MiGRAS* genes were distributed on all scaffolds, but a few scaffolds had multiple *GRAS* genes.

Gene structure analysis showed that 60%, 67.8%, and 53.4% of *MaGRAS, MbGRAS*, and *MiGRAS* genes, respectively, had no intron. *GRAS* genes without intron also occupied a large proportion of other species. For example, 82.2%, 67.6%, 55%, 81.3%, and 77.4% of *Prunus mume (* Lu et al., 2015), *Arabidopsis*, rice (Liu and Widmer, 2014), lychee (Chen et al., 2021), and tomato (Huang et al., 2015), respectively, indicated that *GRAS* family genes were highly conformed in different species. Studies have shown that early eukaryotes had very abundant introns. With the evolution of organisms, most species have fewer introns and only a few species have increased introns. However, most of the introns in the extant species with a high number of introns in their genomes were inherited from their ancestors, with few new additions (Iwamoto et al., 1998). It was speculated that the *GRAS* gene of bananas had intron loss during its evolution. The genes with more introns in bananas mainly exist in the *PAT* and *SCR* subfamilies in *MaGRAS*, *PAT* and *SCR* subfamilies in *MbGRAS*, and *SCR* and *LISCL* subfamilies in *MiGRAS*, suggesting these subfamilies may have had important functions in banana evolution. Functional differences among plant gene family members are regulated to some extent by *cis*-acting elements in their promoter region (Baranwal et al., 2016). Our study found that there are a large number of light responses, phytohormone responses, and stress responses as well as elements related to growth and development in the promoter region of the *GRAS* gene family in bananas. These results indicate that the *GRAS* gene is involved in various stress and hormone responses during banana growth and development, and plays an important role in regulating banana growth at different developmental stages. Many studies have shown that transcription factors play a role in the growth and development of bananas and in banana stress-endurance, for example, bZIP plays an important role in the early development and ripening of bananas (Hu et al., 2016), and C2H2 regulates the ripening of bananas by regulating ethylene synthesis, and regulates cold stress of bananas by inhibiting transcription of ICE1 (Llave et al., 2002; Han and Fu, 2019). There are many kinds of transcription factors in the *GRAS* gene of bananas, and there are 23 kinds of TF in *MaGRAS, MbGRAS*, and *MiGRAS*, indicating that the *GRAS* expression of bananas is regulated by many kinds of transcription factors, which then regulate the growth and development of bananas.

### 4.2 Different gene duplication patterns promote the amplification of the *GRAS* family in bananas

There are some differences in GRAS family phylogenetic relationships among different plants. Studies have shown that the GRAS families of *Arabidopsis* and rice can be attributed to 8 and 14 subfamilies (Tian et al., 2004; Lee et al., 2008), while poplar can be attributed to 13 subfamilies (Liu and Widmer, 2014). The clustering analysis of the GRAS family members of banana, *Arabidopsis*, and rice in the phylogenetic tree showed that the GRAS family in bananas can be classified into 10 subfamilies, indicating that the GRAS family may have acquired new functions during evolution. Combining phylogenetic tree and miRNA prediction analysis, we noted that seven *MaGRAS* genes regulated by miR171 belong to the *HAM* subfamily and have high homology with *AtHAM1, AtHAM2, AtHAM3*, *AtSCL15*, and *AtSCL26* in *Arabidopsis*. Previous studies have shown that *AtHAM1 (AtSCL27)*, *AtHAM2 (AtSCL22*), and *AtHAM3* (*AtSCL6*) are involved in the development of apical meristem and axillary meristem of *Arabidopsis (* Engstrom et al., 2011), and similar effects have been found in petunia (Stuurman et al., 2002). The results of qRT-PCR showed that the expression levels of these seven members were different in different tissues. The expression levels of seven members in pseudostems and leaves were higher than that in roots, and the expression levels of five members in leaves were the highest. It is suggested that the seven *MaGRAS* genes belonging to the *HAM* branch may play an important role in regulating the growth and development of bananas amid environmental stress, and that, except for *MaSCL22*, the other six members of these seven members contain one to two low-temperature response *cis*-acting elements, suggesting that the HAM subfamily may play a role in the process of low-temperature response in bananas.

Whole genome duplication is considered to be the main cause of plant gene family amplification. In the process of evolution, plants form genes with similar functions or structures through gene duplication events (Cannon et al., 2004), and series and fragment duplication genes in plant genomes play an important role in coping with environmental stress (Hanada et al., 2008; D’hont et al., 2012). Studies have found that *GRAS* gene family tandem repeat events are relatively common, with about 2 to 6 tandem repeat genes in each cluster, which occurred twice (4 genes), 12 times (40 genes), 7 times (15 genes), and 4 times (8 genes) in *Arabidopsis*, poplar, rice, and pepper, respectively (Liu and Widmer, 2014; Zhang et al., 2017). In contrast, the occurrence frequency of tandem repeats in the banana *GRAS* gene family was less, with only two tandem repeats in *MaGRAS* and *MiGRAS* (4 genes), and only one tandem repeats in *MiGRAS* (2 genes), representing short clusters. It is speculated that these genes may be the product of the banana’s long evolutionary process and that they play an important role in its reproduction. It has been described that bananas may have undergone three genome-wide duplication events during their evolution, namely, α, β (approximately 75 million years ago), and γ (approximately 100 million years ago) (Zhu et al., 2013). The duplications of the *MaGRAS* family occurred 39.3333–93.4333 million years ago, corresponding to the α or β event. In *MbGRAS* and *MiGRAS*, the duplication events of *MbSCL9-1* and *MiSCL9-1* occurred approximately 268.5561 and 360.9675 million years ago, respectively, corresponding to γ events, while the duplication events of other genes occurred later than 100 million years ago, corresponding to α or β events. It is speculated that whole-genome duplication, gene tandem, and segmental duplication all promoted the *GRAS* gene family amplification. The duplication event of *SCL9-1* occurred earliest in *MaGRAS*, *MbGRAS*, and *MiGRAS*, suggesting that this gene may be originally present in the *GRAS* family of bananas, rather than produced by subsequent gene duplication. In addition, selection pressure analysis revealed that all banana *GRAS* duplication genes underwent purifying selection, suggesting that the family may be functionally conserved.

### 4.3 *GRAS* gene family may be involved in the cold resistance response in bananas

According to promoter identification, nearly half of the *GRAS* genes in bananas have a low-temperature response element, which is not active under normal conditions but can be activated when plants are subjected to low-temperature stress, and plays an important role in plant cold resistance response. In addition, six members of the banana *GRAS* family were found to be regulated by miR171. Plant miRNAs are widely involved in the regulation network of response to low-temperature stress (Zhang et al., 2009; Ye, 2013), and miR171 is a class of low-temperature responsive miRNAs, which are upregulated in *Arabidopsis* and downregulated in rice and *Populus trichocarpa* in response to low-temperature stress (Liu et al., 2008; Lv et al., 2010). MiR171 in *Arabidopsis* affects flower and root development, GA response, light signal transduction, lateral organ polarity, meristem formation sieve tube development and response to adversity, by regulating *SCL6-II(SCL27)*, *SCL6-III(SCL22)*, and *SCL6-IV(SCL6)* of the *GRAS* family (Llave et al., 2002; Wang et al., 2010). The miR171 family of plants is highly conservative, so it is possible that miR171 in bananas may be involved in regulating the hypothermia response mechanism. The analysis found that *MaGRAS* were targeted by multiple members of the miR171 family, suggesting that they may play a role in the banana cold-resistance response. In addition, *GRAS* is involved in the response of various plants to stress. Ye Jiejun (Ye, 2013) found that *GRAS* gene expression in citrus plants was significantly upregulated under low-temperature stress, and the increase of *GRAS* gene expression was positively correlated with plant resistance to low temperature. Guo Peng et al (Guo et al., 2013). used Northern hybridization to study the expression characteristics of *ZmSCL7* in maize under 4 °C stress, and the results showed that *ZmSCL7* was significantly induced by low temperature. *LaSCL18* can actively respond to low-temperature stress, and its downregulated expression trend appeared after 12 h of low-temperature treatment but was always higher than the control level, indicating that this gene may be related to the low-temperature tolerance of *LaSCL18* (Li LL et al., 2017). By analyzing *GRAS* gene expression in ‘Tianbaojiao’ transplants during low-temperature stress, it was found that the *MaGRAS* gene expression pattern during low-temperature stress was not a simple linear change relationship, but a response of multi-directional regulation. *MaSCL27-2, MaSCL27-3*, and *MaSCL6-1* showed negative regulation in response to low temperature, while the other four members showed intermittent positive regulation. In particular, the expression level of *MaSCL27-4* reached nearly eight times that of the control after 24 h of low-temperature treatment, and promoter prediction analysis found that *MaSCL27-4* contained two low-temperature response *cis*-acting elements, suggesting that *MaSCL27-4* may play a major role in the process of low-temperature response in bananas. Therefore, the *GRAS* family of bananas may participate in the cold-resistance response of bananas and play an important role in the cold-resistance process.

In general, miRNAs mainly exist in the form of single chains, which are not completely complementary to the binding region of target mRNA in action, resulting in mismatch, inhibition of translation or degradation of mRNA, and regulation of gene expression at a post-transcriptional level without affecting the stability of mRNA. The online prediction results of miRNA showed that miR171 and its target *GRAS* binding region were completely complementary and paired, which was very similar to the way and function of siRNA degrading target mRNA by means of RNAi. This was consistent with the results of Llave et al., which showed that miR171 and *GRAS* internal sequences of *Arabidopsis* were completely complementary. The RLM-RACE verification results showed a clear targeting relationship between miR171 and *MaGRAS*, which was basically consistent with the software prediction results and research reports. Further analysis of the regulation mode of miR171 and its target genes under low-temperature stress showed that *MaSCL22, MaSCL6-1*, and *MaSCL27-4* were negatively correlated with miR171. These miRNAs regulate the post-transcriptional level expression of the three *MaGRAS* members in response to low-temperature stress by cleaving target mRNA. The response patterns of the other four members were not completely negatively correlated with miR171, but significantly inhibited each other in the early stage, while there was a certain degree of synergistic and complementary inhibition in the later stage, which fully indicated the complexity of post-transcriptional regulation of miRNA and mRNA in response to low-temperature stress in plants. The regulatory functions of miRNA and the regulatory modes of target genes are diverse.

## 5 Conclusion

In this study, a total of 73 *MaGRAS*, 59 *MbGRAS*, and 58 *MiGRAS* genes were identified based on the genomic data of *Musa acuminata*, *M. balbisiana*, and *M. itinerans*. The banana *GRAS* gene promoter has more light, hormone, and stress response elements, indicating that this family may have an important role in plant growth and development and in stress endurance. MiR171 targets seven *MaGRAS* genes and there are differences in the mode of regulation of these seven genes by miR171. In addition, tissue-specific expression analysis showed that the expression of all seven *MaGRAS* regulated by miR171 was downregulated in ‘Tianbaojiao’ transplants at 8 h of low-temperature treatment compared with the 28 °C control, indicating that low-temperature stress affects the expression of *GRAS* genes, which in turn may have important effects on the growth and development of banana and fruit set. In this study, we investigated the regulatory mode of miR171 and its target gene *MaGRAS*, and, in future research, we will further analyze how miR171 and *MaGRAS* genes interact to regulate the resistance of bananas to cold temperatures.

## Data availability statement

The original contributions presented in the study are included in the article/**Supplementary Material**. Further inquiries can be directed to the corresponding author.

## Author contributions

ZL, NT, and DL designed and coordinated the study. NT and DL conducted the experiment and wrote the research paper. DL and SZ prepared the materials and helped complete experiments. NT analyzed the data. MT drew out the pictures. ZL revised the research paper. All authors agree to be accountable for all aspects of the study in ensuring that questions related to the accuracy or integrity of any part of the research work are appropriately investigated and resolved. All authors contributed to the article and approved the submitted version.
