# Supplementary material for: Genome-wide identification and expression analysis of the GRAS family under low-temperature stress in bananas
Source: Front Plant Sci. 2023 Aug 30;14:1216070. doi: 10.3389/fpls.2023.1216070 (PMC10502232; doi:10.3389/fpls.2023.1216070)
Supplement: Supplementary file 1 [file Table_1.docx]

Supplementary Material

**Genome identification and expression analysis under low temperature stress of *GRAS* family in banana**

Ning Tong^#^, Dan Li^#^, Shuting Zhang, Mengjie Tang, Yukun Chen, Zihao Zhang, Yuji Huang, Yuling Lin, Zhenguang Cheng, Zhongxiong Lai^*^

Institute of Horticultural Biotechnology, Fujian Agriculture and Forestry University, Fuzhou, 350002, China

^#^ These authors contributed equally to this work.

^*^Corresponding author.

E-mail address: laizx01@163.com (Z. Lai).

**Supplementary Data**

**Supplementary Table 1.**The informations of *GRAS* gene family.

|  | Gene ID | Gene name | Chr location | amino acids | bp | MW | pI | Instability index |
| --- | --- | --- | --- | --- | --- | --- | --- | --- |
| Musa acuminata | *Ma00_g04570* | *MaSCL9-5* | chr0:39138536..39141564 | 804 | 2415 | 90217.59 | 5.68 | 46.1 |
|  | *Ma01_g00100* | *MaSCL18-1* | chr1:98764..100586 | 435 | 1308 | 48200.22 | 6.3 | 57.67 |
|  | *Ma01_g09550* | *MaSCL28-1* | chr1:6871795..6873489 | 510 | 1533 | 56600.09 | 5.63 | 65.74 |
|  | *Ma01_g12080* | *MaSCL28-2* | chr1:8790351..8793133 | 614 | 1845 | 67549.15 | 5.55 | 59.42 |
|  | *Ma01_g12700* | *MaSCL18-2* | chr1:9306243..9309797 | 489 | 1470 | 54187.52 | 6.26 | 52.19 |
|  | *Ma01_g14650* | *MaSLR1-1* | chr1:10703439..10705983 | 643 | 1932 | 68927.84 | 5.87 | 50.8 |
|  | *Ma01_g15900* | *MaSHR1-1* | chr1:11583852..11585836 | 532 | 1599 | 58658.01 | 5.42 | 67.46 |
|  | *Ma01_g22010* | *MaCIGR2-1* | chr1:22594473..22598410 | 536 | 1611 | 60248.9 | 5.67 | 53.83 |
|  | *Ma02_g24350* | *MaSLR1-2* | chr2:29012993..29015431 | 612 | 1839 | 65708.15 | 5.67 | 47.05 |
|  | *Ma03_g02280* | *MaSCL1-1a* | chr3:1548680..1555566 | 587 | 1764 | 65035.32 | 4.93 | 45.63 |
|  |  | *MaSCL1-1b* |  | 568 | 1707 | 63190.12 | 4.95 | 49.43 |
|  |  | *MaSCL1-1c* |  | 554 | 1665 | 61530.15 | 4.79 | 51.01 |
|  | *Ma03_g11670* | *MaSCL18-3* | chr3:9063300..9064619 | 411 | 1236 | 46042.6 | 6.08 | 55.61 |
|  | *Ma03_g31190* | *MaSCL15-1* | chr3:33493550..33495523 | 531 | 1596 | 56224.4 | 5.73 | 50.51 |
|  | *Ma03_g31740* | *MaSCL4-1* | chr3:33839235..33842715 | 634 | 1905 | 68440.57 | 5.02 | 54.29 |
|  | *Ma04_g03560* | *MaSCL15-2* | chr4:2728596..2730801 | 534 | 1605 | 56728.63 | 6.17 | 52.97 |
|  | *Ma04_g07000* | *MaSCL21* | chr4:5087433..5091012 | 539 | 1620 | 60640.56 | 5.96 | 56.15 |
|  | *Ma04_g07170* | *MaSCL15-3* | chr4:5218667..5220589 | 522 | 1569 | 56246.64 | 6.11 | 50.59 |
|  | *Ma04_g07320* | *MaSCL4-2* | chr4:5314074..5316835 | 621 | 1866 | 67316.18 | 5.35 | 58.06 |
|  | *Ma04_g11040* | *MaCIGR2-2* | chr4:7778036..7781022 | 515 | 1548 | 57358.82 | 5.4 | 49.69 |
|  | *Ma04_g17020* | *MaSHR1-2* | chr4:17237911..17239861 | 531 | 1596 | 58713.54 | 6.53 | 57.8 |
|  | *Ma04_g23420* | *MaSCL8-1* | chr4:25593128..25595314 | 591 | 1776 | 62982.46 | 8.53 | 58.58 |
|  | *Ma04_g25650* | *MaSCL6-1* | chr4:27365241..27368813 | 726 | 2181 | 78025.62 | 6.16 | 56.41 |
|  | *Ma04_g26390* | *MaSCL28-3* | chr4:27860930..27863618 | 646 | 1941 | 70970.83 | 6.03 | 67.25 |
|  | *Ma04_g30070* | *MaSCL23-1* | chr4:30822158..30824374 | 465 | 1398 | 50459.26 | 6.03 | 51.86 |
|  | *Ma04_g31520* | *MaSCL8-2* | chr4:31797697..31799808 | 575 | 1728 | 61116.68 | 6.32 | 56.82 |
|  | *Ma04_g32970* | *MaSCL8-3* | chr4:32834346..32836561 | 584 | 1755 | 62101.33 | 8.92 | 52.7 |
|  | *Ma05_g08020* | *MaSCL14* | chr5:5960201..5963438 | 684 | 2055 | 77116.34 | 5.26 | 56.33 |
|  | *Ma05_g12570* | *MaSCL8-4* | chr5:9086765..9088860 | 584 | 1755 | 62119.23 | 8.12 | 56.67 |
|  | *Ma05_g16130* | *MaSCL8-5* | chr5:15331061..15333220 | 585 | 1758 | 62377.58 | 6.67 | 51.01 |
|  | *Ma05_g23270* | *MaSCL27-1* | chr5:35304861..35310175 | 784 | 2355 | 84988.75 | 6.29 | 58.8 |
|  | *Ma05_g23910* | *MaSCR-1* | chr5:36092199..36095880 | 802 | 2409 | 85993.61 | 5.66 | 67 |
|  | *Ma06_g09430* | *MaSCL32-1* | chr6:6610029..6611865 | 457 | 1374 | 50287.52 | 5.33 | 52.83 |
|  | *Ma06_g13300* | *MaSCL1-2* | chr6:9110523..9115149 | 579 | 1740 | 64446.71 | 5.07 | 47.77 |
|  | *Ma06_g18350* | *MaSCL32-2* | chr6:12469365..12471271 | 457 | 1374 | 50473.04 | 5.4 | 49.58 |
|  | *Ma06_g19260* | *MaSCL23-2* | chr6:13231445..13233521 | 446 | 1341 | 49001.68 | 6.34 | 43.49 |
|  | *Ma06_g22250* | *MaGAI-1* | chr6:17603070..17605383 | 726 | 2181 | 79481.75 | 6.13 | 60.22 |
|  | *Ma06_g25650* | *MaSCL32-3* | chr6:26033838..26035720 | 572 | 1332 | 64044.03 | 6.43 | 50.1 |
|  | *Ma06_g25670* | *MaCIGR1-1* | chr6:26040398..26045427 | 443 | 1719 | 48912.47 | 5.36 | 42.12 |
|  | *Ma06_g36050* | *MaSCL9-1* | chr6:35665059..35668124 | 794 | 2385 | 88698.24 | 5.52 | 49.38 |
|  | *Ma06_g36470* | *MaGAI-2* | chr6:35868472..35870468 | 608 | 1827 | 66115.11 | 5.78 | 43.92 |
|  | *Ma06_g38750* | *MaSCL22* | chr6:37388533..37390802 | 486 | 1461 | 53975.99 | 8.61 | 52.19 |
|  | *Ma07_g05950* | *MaSCL28-4a* | chr7:4299770..4302929 | 616 | 1851 | 68253.28 | 6.09 | 60.94 |
|  |  | *MaSCL28-4b* |  | 650 | 1953 | 71598.6 | 5.96 | 62.54 |
|  | *Ma07_g06770* | *MaSCL6-2* | chr7:4861964..4864609 | 718 | 2157 | 77436.1 | 5.92 | 62.7 |
|  | *Ma07_g15010* | *MaSCL9-2* | chr7:11265912..11269197 | 748 | 2247 | 84098.71 | 6.54 | 49.89 |
|  | *Ma07_g16700* | *MaSCL18-4* | chr7:15233941..15235155 | 404 | 1215 | 45126.97 | 6.19 | 53.38 |
|  | *Ma07_g28380* | *MaSCL4-3* | chr7:34460385..34462432 | 621 | 1866 | 67370.85 | 4.84 | 45.71 |
|  | *Ma08_g01100* | *MaRGA2* | chr8:1007125..1008730 | 510 | 1533 | 56583.72 | 5.89 | 52.06 |
|  | *Ma08_g02350* | *MaSCL33* | chr8:1848789..1852272 | 805 | 2418 | 90003.95 | 5.44 | 50.82 |
|  | *Ma08_g03530* | *MaCIGR1-2* | chr8:2558540..2566293 | 558 | 1677 | 62682.4 | 5.68 | 43.94 |
|  | *Ma08_g09130* | *MaSCR-2a* | chr8:6600964..6604213 | 712 | 2139 | 75756.45 | 5.61 | 51.1 |
|  |  | *MaSCR-2b* |  | 710 | 2133 | 75705.49 | 5.83 | 54.2 |
|  |  | *MaSCR-2c* |  | 699 | 2100 | 74298.89 | 5.75 | 51.48 |
|  | *Ma08_g09470* | *MaSCL3-1* | chr8:6854895..6857266 | 474 | 1425 | 52799.41 | 5.91 | 55.04 |
|  | *Ma08_g10950* | *MaSCL27-2* | chr8:8082512..8085882 | 786 | 2361 | 85207.54 | 6.21 | 48.91 |
|  | *Ma08_g16560* | *MaNSP2* | chr8:19228898..19230691 | 529 | 1590 | 56975.22 | 5.17 | 48.43 |
|  | *Ma08_g17700* | *MaSLR1-3* | chr8:26783151..26785802 | 605 | 1818 | 65631.04 | 5.26 | 47.57 |
|  | *Ma08_g31390* | *MaSHR1-3* | chr8:42318304..42320576 | 530 | 1593 | 58864.34 | 5.47 | 63.56 |
|  | *Ma09_g02320* | *MaSHR2-1* | chr9:1652601..1653830 | 409 | 1230 | 46363.62 | 5.02 | 48.7 |
|  | *Ma09_g11650* | *MaCIGR1-3* | chr9:7889509..7893561 | 570 | 1713 | 63734.05 | 5.29 | 50.82 |
|  | *Ma09_g19630* | *MaSCL8-6* | chr9:25388014..25389983 | 569 | 1710 | 60757.32 | 6.75 | 53.66 |
|  | *Ma09_g20690* | *MaSHR2-2* | chr9:29865464..29866715 | 401 | 1206 | 45328.46 | 5.26 | 52.36 |
|  | *Ma09_g25150* | *MaCIGR1-4* | chr9:36706720..36711859 | 464 | 1395 | 52151.76 | 8.39 | 44.74 |
|  | *Ma10_g02270* | *MaSCL9-3* | chr10:7571817..7575256 | 750 | 2253 | 84129.66 | 6.5 | 53.69 |
|  | *Ma10_g10710* | *MaSCL23-3* | chr10:24508117..24509560 | 449 | 1350 | 50257.18 | 8.6 | 48.5 |
|  | *Ma10_g11650* | *MaSCL32-4* | chr10:25101598..25103527 | 455 | 1368 | 50353.73 | 5.68 | 52.81 |
|  | *Ma10_g16450* | *MaSHR2-3* | chr10:28149290..28150498 | 402 | 1209 | 44902.63 | 5.33 | 43.22 |
|  | *Ma10_g17350* | *MaSCL1-3* | chr10:28790199..28796504 | 586 | 1761 | 65008.11 | 4.99 | 46.13 |
|  | *Ma10_g17600* | *MaNSP1* | chr10:28942684..28945333 | 530 | 1593 | 57492.32 | 6.24 | 42.92 |
|  | *Ma10_g19630* | *MaSLN1* | chr10:30140087..30142521 | 595 | 1788 | 63810.07 | 5.2 | 46.88 |
|  | *Ma10_g22580* | *MaSCL9-4* | chr10:31897640..31901515 | 760 | 2283 | 84855.2 | 7.59 | 56.1 |
|  | *Ma11_g02690* | *MaSCL8-7* | chr11:1975330..1977447 | 580 | 1743 | 62087.42 | 8.87 | 56.28 |
|  | *Ma11_g04080* | *MaSCL27-3* | chr11:3178354..3181614 | 790 | 2373 | 85301.71 | 6.03 | 51.69 |
|  | *Ma11_g05610* | *MaSCL3-2* | chr11:4307844..4309885 | 473 | 1422 | 52700.31 | 6.22 | 52.95 |
|  | *Ma11_g18500* | *MaSCR-3* | chr11:23544904..23548184 | 783 | 2352 | 83801.52 | 5.67 | 66.65 |
|  | *Ma11_g18690* | *MaSCL3-3a* | chr11:23715638..23718247 | 463 | 1392 | 51501.31 | 6.48 | 53.35 |
|  |  | *MaSCL3-3b* |  | 476 | 1431 | 52884.92 | 6.77 | 54.4 |
|  | *Ma11_g19440* | *MaSCL27-4* | chr11:24298407..24301624 | 782 | 2349 | 84888.26 | 5.51 | 49.99 |
| Musa balbisiana | *Mba01_g01860* | *MbSCL4-1* | chr1:1247347..1249059 | 570 | 1713 | 61098.44 | 4.8 | 49.68 |
|  | *Mba01_g19140* | *MbSCL28-1* | chr1:14738161..14739855 | 514 | 1545 | 57369.34 | 6.01 | 64.8 |
|  | *Mba01_g22320* | *MbSCL18-1* | chr1:17257343..17259734 | 615 | 1848 | 67141.15 | 6.58 | 58.98 |
|  | *Mba01_g24230* | *MbSLR1-1* | chr1:18684970..18686877 | 635 | 1908 | 68146.81 | 5.67 | 51.7 |
|  | *Mba01_g31370* | *MbSHR1-1* | chr1:39450874..39452502 | 542 | 1629 | 59452.87 | 5.61 | 60.47 |
|  | *Mba02_g23160* | *MbSLR1-2* | chr2:32271462..32273303 | 613 | 1842 | 65869.57 | 5.81 | 46.89 |
|  | *Mba03_g02150* | *MbSCL1-1* | chr3:1660322..1670768 | 601 | 1806 | 66712.31 | 4.98 | 46.73 |
|  | *Mba03_g11270* | *MbSCL18-2* | chr3:9224066..9225424 | 452 | 1359 | 50377.6 | 6.01 | 58.68 |
|  | *Mba03_g22670* | *MbSCL18-3* | chr3:30369843..30371150 | 435 | 1308 | 48073.2 | 6.36 | 57.6 |
|  | *Mba04_g03410* | *MbSCL15-1* | chr4:3016548..3019454 | 566 | 1701 | 60092.67 | 6.26 | 54.12 |
|  | *Mba04_g06670* | *MbSCL21* | chr4:5448677..5452104 | 607 | 1824 | 68375.67 | 6.56 | 55.59 |
|  | *Mba04_g06850* | *MbSCL15-2* | chr4:5581642..5583210 | 522 | 1569 | 56392.52 | 5.74 | 48.53 |
|  | *Mba04_g06990* | *MbSCL4-2* | chr4:5677788..5679341 | 517 | 1554 | 55971.71 | 5.24 | 55.07 |
|  | *Mba04_g10680* | *MbCIGR2* | chr4:8321859..8323193 | 444 | 1335 | 49631.63 | 5.6 | 42.04 |
|  | *Mba04_g16510* | *MbSCL4-3* | chr4:17472352..17473422 | 356 | 1071 | 37670.47 | 4.95 | 58.54 |
|  | *Mba04_g17680* | *MbSHR1-2* | chr4:21465809..21467398 | 529 | 1590 | 58621.56 | 7.02 | 58.9 |
|  | *Mba04_g24120* | *MbSCL8-1* | chr4:30697144..30698556 | 470 | 1413 | 50123.82 | 6.4 | 56.09 |
|  | *Mba04_g26250* | *MbSCL6-1* | chr4:32564507..32566708 | 733 | 2202 | 78642.32 | 6.12 | 57.4 |
|  | *Mba04_g26950* | *MbSCL28-2* | chr4:33052122..33052122 | 489 | 1470 | 55710.76 | 5.17 | 64.86 |
|  | *Mba04_g30840* | *MbSCL23-1* | chr4:36226697..36228094 | 465 | 1398 | 50344.12 | 5.96 | 51.67 |
|  | *Mba04_g32320* | *MbSCL8-2* | chr4:37227390..37229117 | 575 | 1728 | 61182.69 | 6.16 | 54.42 |
|  | *Mba04_g33600* | *MbSCL8-3* | chr4:38173881..38175644 | 587 | 1764 | 62440.78 | 8.74 | 53 |
|  | *Mba05_g05110* | *MbSCL8-4* | chr5:4156874..4158340 | 488 | 1467 | 51917.76 | 6.19 | 50.85 |
|  | *Mba05_g15340* | *MbSCL8-5* | chr5:13939070..13942982 | 831 | 2496 | 87816.34 | 6.47 | 51.7 |
|  | *Mba05_g22880* | *MbSCL27-1* | chr5:35596812..35599166 | 784 | 2355 | 84699.35 | 6.21 | 58.15 |
|  | *Mba05_g23530* | *MbSCR-1* | chr5:36371745..36381335 | 838 | 2517 | 90301.62 | 6.38 | 66.41 |
|  | *Mba06_g12400* | *MbSCL1-2* | chr6l9594136..9598857 | 579 | 1740 | 64324.62 | 5.06 | 46.97 |
|  | *Mba06_g17340* | *MbSCL32-1* | chr6:12970362..12971735 | 457 | 1374 | 50656.07 | 5.19 | 54.45 |
|  | *Mba06_g20740* | *MbGAI* | chr6:17216153..17219624 | 769 | 2310 | 83879.56 | 5.85 | 57.74 |
|  | *Mba06_g21600* | *MbSHR2-1* | chr6:19303297..19304502 | 401 | 1206 | 45272.48 | 5.33 | 53.39 |
|  | *Mba06_g25020* | *MbCIGR1-1* | chr6:30209567..30222992 | 991 | 2976 | 108846.87 | 7.75 | 46.36 |
|  | *Mba06_g34910* | *MbSCL9-1* | chr6:39310641..39313028 | 795 | 2388 | 88808.54 | 5.48 | 48.3 |
|  | *Mba06_g37370* | *MbSCL6-2* | chr6:41238341..41240533 | 730 | 2193 | 78589.54 | 5.77 | 52.93 |
|  | *Mba07_g06350* | *MbSCL6-3* | chr7:5350204..5352348 | 714 | 2145 | 76940.77 | 5.87 | 62.65 |
|  | *Mba07_g13640* | *MbSCL9-2* | chr7:11952591..11954837 | 748 | 2247 | 84209.65 | 6.19 | 50.12 |
|  | *Mba07_g16060* | *MbSCL18-4* | chr7:21391806..21393020 | 404 | 1215 | 45060.87 | 6.19 | 53.95 |
|  | *Mba07_g26490* | *MbSCL4-4* | chr7:36246742..36248796 | 550 | 1653 | 59411.73 | 4.76 | 42.81 |
|  | *Mba08_g01110* | *MbRGA2* | chr8:1098862..1100634 | 464 | 1395 | 51422.71 | 5.64 | 51.46 |
|  | *Mba08_g03240* | *MbCIGR1-2* | chr8:2696340..2705813 | 594 | 1785 | 66835.99 | 5.38 | 46.61 |
|  | *Mba08_g08700* | *MbSCR-2* | chr8:6995700..6999523 | 710 | 2133 | 75618.41 | 5.76 | 54.5 |
|  | *Mba08_g09050* | *MbSCL3-1* | chr8:7267752..7269176 | 474 | 1425 | 52787.43 | 5.91 | 56.95 |
|  | *Mba08_g10500* | *MbSCL27-2* | chr8:8535193..8537553 | 786 | 2361 | 85088.38 | 6.09 | 49.68 |
|  | *Mba08_g16250* | *MbNSP2* | chr8:20225989..20227578 | 529 | 1590 | 57147.37 | 5.16 | 45.28 |
|  | *Mba08_g30940* | *MbSHR1-3* | chr8:42618806..42620389 | 527 | 1584 | 58637.12 | 5.45 | 64.49 |
|  | *Mba09_g02280* | *MbSHR2-2* | chr9:1830683..1831912 | 409 | 1230 | 46394.66 | 5.02 | 49.45 |
|  | *Mba09_g11100* | *MbCIGR1-3* | chr9:8025905..8027617 | 570 | 1713 | 63859.26 | 5.29 | 51.13 |
|  | *Mba09_g18250* | *MbSCL8-6* | chr9:17965988..17967706 | 572 | 1719 | 60914.44 | 6.26 | 52.3 |
|  | *Mba10_g02800* | *MbSCL4-5* | chr10:12687091..12688041 | 316 | 951 | 33149.59 | 4.43 | 53.97 |
|  | *Mba10_g09340* | *MbSCL23-2* | chr10:28317902..28319221 | 439 | 1320 | 49175.67 | 6.87 | 45.2 |
|  | *Mba10_g10170* | *MbSCL32-2* | chr10:28905958..28907157 | 399 | 1200 | 44207.73 | 5.28 | 50.94 |
|  | *Mba10_g14390* | *MbSHR2-3* | chr10:32020218..32022891 | 420 | 1263 | 47028.07 | 5.64 | 40.77 |
|  | *Mba10_g15260* | *MbSCL1-3* | chr10:32652109..32658379 | 705 | 2118 | 78546.12 | 5.35 | 48.64 |
|  | *Mba10_g15470* | *MbNSP1* | chr10:32818860..32820452 | 530 | 1593 | 57555.37 | 6.2 | 43.65 |
|  | *Mba10_g17320* | *MbSLN1* | chr10:34120054..34121841 | 595 | 1788 | 63693.07 | 5.25 | 47.9 |
|  | *Mba10_g21390* | *MbSCL4-6* | chr10:37077899..37078978 | 359 | 1080 | 37215.79 | 6.48 | 52.45 |
|  | *Mba11_g02550* | *MbSCL8-7* | chr11:2246881..2248620 | 579 | 1740 | 62036.44 | 8.87 | 54.76 |
|  | *Mba11_g04000* | *MbSCL27-3* | chr11:3559508..3564868 | 829 | 2490 | 89646.72 | 6.12 | 48.54 |
|  | *Mba11_g05450* | *MbSCL3-2* | chr11:4917978..4919399 | 473 | 1422 | 52592.16 | 6.2 | 50.45 |
|  | *Mba11_g17760* | *MbSCR-3* | chr11:24526027..24529243 | 785 | 2358 | 83932.64 | 5.71 | 66.99 |
| Musa itinerans | *Mi_g000789* | *MiSCL18-4* | scaffold1037:123231..124325 | 365 | 1095 | 41052.51 | 6.21 | 55.77 |
|  | *Mi_g001848* | *MiSCL8-1* | scaffold1140:81131..82894 | 587 | 1764 | 62500.82 | 8.73 | 49.99 |
|  | *Mi_g002329* | *MiSHR1-2* | scaffold1167:789616..791202 | 528 | 1587 | 58707.17 | 5.36 | 65.63 |
|  | *Mi_g003925* | *MiSCL15-2* | scaffold1329:74666..76264 | 532 | 1599 | 56396.31 | 6.01 | 55.06 |
|  | *Mi_g004093* | *MiSLN1* | scaffold1336:46377..48164 | 489 | 1470 | 52042.08 | 5.8 | 42.29 |
|  | *Mi_g004252* | *MiGAI-1* | scaffold1345:531951..533852 | 595 | 1785 | 65186.03 | 6.11 | 47.24 |
|  | *Mi_g004290* | *MiSCL9-1* | scaffold1345:750995..753160 | 612 | 1835 | 68966.99 | 5.93 | 48 |
|  | *Mi_g004371* | *MiSCL32-1* | scaffold1353:134842..136215 | 457 | 1374 | 50382.73 | 5.28 | 54.49 |
|  | *Mi_g004538* | *MiSHR2-2* | scaffold1369:17548..18672 | 375 | 1125 | 42334.32 | 5.45 | 54.45 |
|  | *Mi_g004915* | *MiSCL6-1* | scaffold1408:250953..253067 | 618 | 1854 | 65876.83 | 5.58 | 52.9 |
|  | *Mi_g004991* | *MiSCL8-3* | scaffold1413:196870..198624 | 584 | 1755 | 61944.12 | 8.12 | 57.29 |
|  | *Mi_g005751* | *MiRGA2* | scaffold1489:231736..233268 | 457 | 1374 | 51021.11 | 5.8 | 54.43 |
|  | *Mi_g005822* | *MiSCL9-4* | scaffold1511:94051..96719 | 678 | 2034 | 76592.42 | 5.77 | 47.07 |
|  | *Mi_g006626* | *MiSCR-3* | scaffold1584:147543..149150 | 382 | 1146 | 42068.94 | 5.91 | 45.37 |
|  | *Mi_g006646* | *MiSCL3-3* | scaffold1584:338631..343184 | 617 | 1851 | 69258.86 | 6.48 | 51.03 |
|  | *Mi_g007400* | *MiSCL28-2* | scaffold1664:348239..350191 | 650 | 1953 | 71733.77 | 6.03 | 62.11 |
|  | *Mi_g008072* | *MiSCL18-3* | scaffold1750:95184..96407 | 408 | 1224 | 45190.02 | 6.38 | 55.14 |
|  | *Mi_g008319* | *MiCIGR1-3* | scaffold179:2994..9163 | 579 | 1737 | 65204.99 | 9.14 | 58.51 |
|  | *Mi_g008490* | *MiSCL28-4* | scaffold1812:317806..319653 | 615 | 1848 | 67447.9 | 5.62 | 62.1 |
|  | *Mi_g008550* | *MiSCL6-2* | scaffold1819:36909..39044 | 664 | 1992 | 71800.75 | 5.75 | 59.11 |
|  | *Mi_g009488* | *MiSCL18-1* | scaffold1917:64346..65539 | 398 | 1194 | 44592.4 | 6.73 | 52.24 |
|  | *Mi_g011314* | *MiSHR2-3* | scaffold216:291851..292864 | 338 | 1014 | 37943.74 | 5.52 | 47.18 |
|  | *Mi_g012925* | *MiSCL27-2* | scaffold239:351378..353726 | 693 | 2082 | 75683.65 | 5.66 | 46.32 |
|  | *Mi_g012984* | *MiSCL32-2* | scaffold2393:181492..182736 | 415 | 1245 | 45998.73 | 5.3 | 53.16 |
|  | *Mi_g013550* | *MiGAI-2* | scaffold2484:27241..29475 | 610 | 1833 | 66452.52 | 5.49 | 52.74 |
|  | *Mi_g014628* | *MiSCL32-4* | scaffold2627:580429..581805 | 459 | 1377 | 50869.4 | 5.59 | 51.36 |
|  | *Mi_g015188* | *MiSCL1-3* | scaffold2710:213120..215201 | 578 | 1734 | 64352.46 | 5.03 | 47.16 |
|  | *Mi_g015524* | *MiSCL23-1* | scaffold275:451253..452494 | 414 | 1242 | 45716.88 | 6.21 | 42.25 |
|  | *Mi_g016837* | *MiSHR2-1* | scaffold2934:46405..47529 | 375 | 1124 | 42392.23 | 5.14 | 47.27 |
|  | *Mi_g017201* | *MiSCL1-2* | scaffold3004:92969..95162 | 586 | 1761 | 64858.92 | 4.91 | 47.29 |
|  | *Mi_g017221* | *MiNSP1* | scaffold3004:254493..256085 | 447 | 1344 | 48547.31 | 6.53 | 42.03 |
|  | *Mi_g017721* | *MiSCR-2* | scaffold31:110453..113221 | 683 | 2052 | 72677.06 | 5.6 | 48.98 |
|  | *Mi_g018131* | *MiSCL9-3* | scaffold320:189898..191940 | 562 | 1686 | 64151.46 | 6.63 | 46.28 |
|  | *Mi_g018272* | *MiSCL8-4* | scaffold3230:16304..18022 | 572 | 1719 | 61074.52 | 6.03 | 52.11 |
|  | *Mi_g018749* | *MiSCL4-1* | scaffold333:432900..434810 | 579 | 1740 | 62209.73 | 4.85 | 51.46 |
|  | *Mi_g018794* | *MiSCL15-1* | scaffold333:805569..807164 | 531 | 1596 | 56173.38 | 5.69 | 51.05 |
|  | *Mi_g019209* | *MiSCL4-2* | scaffold3449:61339..63210 | 587 | 1764 | 64186.15 | 5.06 | 45.67 |
|  | *Mi_g020854* | *MiSCL9-2* | scaffold3946:112125..114305 | 624 | 1872 | 70315.31 | 7.32 | 51.81 |
|  | *Mi_g021121* | *MiSCL1-1* | scaffold400:596361..598658 | 586 | 1761 | 64905.08 | 4.9 | 46.7 |
|  | *Mi_g022287* | *MiSCL3-2* | scaffold4277:10155..11576 | 473 | 1422 | 52629.22 | 6.07 | 51.78 |
|  | *Mi_g023504* | *MiSHR1-1* | scaffold477:229642..231237 | 531 | 1596 | 58581.94 | 5.35 | 67.58 |
|  | *Mi_g023914* | *MiSCL23-2* | scaffold495:62718..64037 | 439 | 1320 | 48959.42 | 6.68 | 43.37 |
|  | *Mi_g024689* | *MiSCL28-1* | scaffold515:136660..146791 | 888 | 2667 | 98811.03 | 5.76 | 66.09 |
|  | *Mi_g025012* | *MiCIGR2* | scaffold525:126067..127521 | 485 | 1455 | 54023.43 | 5.69 | 46.86 |
|  | *Mi_g025882* | *MiSCL27-3* | scaffold5729:24306..26693 | 707 | 2124 | 76200.27 | 5.79 | 47.27 |
|  | *Mi_g026164* | *MiNSP2* | scaffold5889:21122..22705 | 527 | 1584 | 56880.02 | 5.17 | 44.83 |
|  | *Mi_g027166* | *MiSCL28-3* | scaffold63:64457..66055 | 533 | 1599 | 58518.42 | 5.55 | 65.83 |
|  | *Mi_g027450* | *MiSCL18-2* | scaffold649:327082..328194 | 371 | 1113 | 41437.59 | 5.62 | 51.98 |
|  | *Mi_g027582* | *MiCIGR1-1* | scaffold655:340429..342141 | 570 | 1713 | 63851.24 | 5.33 | 50.47 |
|  | *Mi_g027597* | *MiCIGR1-2* | scaffold658:226287..228002 | 534 | 1605 | 59304.62 | 6.05 | 45.29 |
|  | *Mi_g027598* | *MiSCL32-3* | scaffold658:233192..234514 | 441 | 1323 | 48731.25 | 5.36 | 44.32 |
|  | *Mi_g027691* | *MiSCL8-2* | scaffold6590:54338..56092 | 584 | 1755 | 62373.46 | 6.39 | 53.07 |
|  | *Mi_g028054* | *MiSCR-1* | scaffold676:65858..67582 | 357 | 1071 | 39231.65 | 5.75 | 43.43 |
|  | *Mi_g028399* | *MiSCL27-1* | scaffold698:585892..588252 | 704 | 2115 | 76233.4 | 5.93 | 46.68 |
|  | *Mi_g029746* | *MiSCL4-3* | scaffold784:39257..41113 | 588 | 1764 | 63936.41 | 5.39 | 56.94 |
|  | *Mi_g029761* | *MiSCL15-3* | scaffold784:140573..142138 | 521 | 1566 | 55886.15 | 5.85 | 49.62 |
|  | *Mi_g029776* | *MiSCL21* | scaffold784:293376..294809 | 478 | 1434 | 53417.76 | 5.76 | 53.27 |
|  | *Mi_g030030* | *MiSCL3-1* | scaffold796:7089..8513 | 474 | 1425 | 52735.3 | 5.99 | 57.01 |

**Supplementary Table 2.***GRAS* family gene duplication events in banana.

| Genename | GeneID | Genename | GeneID | Ka | Ks | Ka/Ks | Duplication  date/Mya |
| --- | --- | --- | --- | --- | --- | --- | --- |
| *MaSCL28-1* | *Ma01_t09550.1* | *MaSCL28-2* | *Ma01_t12080.1* | 0.2135 | 0.7303 | 0.2924 | 81.1444 |
| *MaSLR1-1* | *Ma01_t14650.1* | *MaSLR1-2* | *Ma02_t24350.1* | 0.1694 | 0.6622 | 0.2559 | 73.5778 |
| *MaSCL18-2* | *Ma01_t12700.1* | *MaSCL18-3* | *Ma03_t11670.1* | 0.0759 | 0.4040 | 0.1880 | 44.8889 |
| *MaSCL28-2* | *Ma01_t12080.1* | *MaSCL28-3* | *Ma04_t26390.1* | 0.1489 | 0.6153 | 0.2420 | 68.3667 |
| *MaSCL28-2* | *Ma01_t12080.1* | *MaSCL28-4b* | *Ma07_t05950.2* | 0.1309 | 0.6819 | 0.1919 | 75.7667 |
| *MaSHR1-1* | *Ma01_t15900.1* | *MaSHR1-3* | *Ma08_t31390.1* | 0.1072 | 0.4837 | 0.2217 | 53.7444 |
| *MaSLR1-2* | *Ma02_t24350.1* | *MaSLR1-3* | *Ma08_t17700.1* | 0.1003 | 0.4752 | 0.2111 | 52.8000 |
| *MaSLR1-2* | *Ma02_t24350.1* | *MaSLN1* | *Ma10_t19630.1* | 0.0944 | 0.4715 | 0.2003 | 52.3889 |
| *MaSCL4-1* | *Ma03_t31740.1* | *MaSCL4-2* | *Ma04_t07320.1* | 0.1268 | 0.5074 | 0.2498 | 56.3778 |
| *MaSCL15-1* | *Ma03_t31190.1* | *MaSCL15-2* | *Ma04_t03560.1* | 0.1404 | 0.5656 | 0.2482 | 62.8444 |
| *MaSCL4-1* | *Ma03_t31740.1* | *MaSCL4-3* | *Ma07_t28380.1* | 0.1283 | 0.4059 | 0.3162 | 45.1000 |
| *MaSCL8-1* | *Ma04_t23420.1* | *MaSCL8-2* | *Ma04_t31520.1* | 0.1807 | 0.5044 | 0.3583 | 56.0444 |
| *MaSCL15-2* | *Ma04_t03560.1* | *MaSCL15-3* | *Ma04_t07170.1* | 0.1988 | 0.4831 | 0.4116 | 53.6778 |
| *MaSCL8-3* | *Ma04_t32970.1* | *MaSCL8-4* | *Ma05_t12570.1* | 0.1418 | 0.4909 | 0.2889 | 54.5444 |
| *MaSCL8-3* | *Ma04_t32970.1* | *MaSCL8-5* | *Ma05_t16130.1* | 0.1268 | 0.4419 | 0.2870 | 49.1000 |
| *MaSCL6-1* | *Ma04_t25650.1* | *MaSCL22* | *Ma06_t38750.1* | 0.1535 | 0.5764 | 0.2664 | 64.0444 |
| *MaSCL4-2* | *Ma04_t07320.1* | *MaSCL4-3* | *Ma07_t28380.1* | 0.1735 | 0.5352 | 0.3242 | 59.4667 |
| *MaSCL6-1* | *Ma04_t25650.1* | *MaSCL6-2* | *Ma07_t06770.1* | 0.1524 | 0.3603 | 0.4231 | 40.0333 |
| *MaSCL28-3* | *Ma04_t26390.1* | *MaSCL28-4b* | *Ma07_t05950.2* | 0.0862 | 0.4452 | 0.1935 | 49.4667 |
| *MaSCL8-1* | *Ma04_t23420.1* | *MaSCL8-7* | *Ma11_t02690.1* | 0.2602 | 0.8220 | 0.3165 | 91.3333 |
| *MaSCL8-3* | *Ma04_t32970.1* | *MaSCL8-7* | *Ma11_t02690.1* | 0.2361 | 0.8223 | 0.2872 | 91.3667 |
| *MaSCL8-4* | *Ma05_t12570.1* | *MaSCL8-5* | *Ma05_t16130.1* | 0.1549 | 0.5471 | 0.2831 | 60.7889 |
| *MaSCL27-1* | *Ma05_t23270.1* | *MaSCL27-2* | *Ma08_t10950.1* | 0.1540 | 0.5463 | 0.2819 | 60.7000 |
| *MaSCL27-1* | *Ma05_t23270.1* | *MaSCL27-3* | *Ma11_t04080.1* | 0.1385 | 0.5040 | 0.2749 | 56.0000 |
| *MaSCL27-1* | *Ma05_t23270.1* | *MaSCL27-4* | *Ma11_t19440.1* | 0.1474 | 0.4072 | 0.3620 | 45.2444 |
| *MaSCL8-4* | *Ma05_t12570.1* | *MaSCL8-7* | *Ma11_t02690.1* | 0.2335 | 0.7884 | 0.2962 | 87.6000 |
| *MaSCL32-1* | *Ma06_t09430.1* | *MaSCL32-2* | *Ma06_t18350.1* | 0.1159 | 0.4767 | 0.2432 | 52.9667 |
| *MaSCL9-1* | *Ma06_t36050.1* | *MaSCL33* | *Ma08_t02350.1* | 0.1632 | 0.8409 | 0.1940 | 93.4333 |
| *MaCIGR1-1* | *Ma06_t25670.1* | *MaCIGR1-4* | *Ma09_t25150.1* | 0.1024 | 0.6001 | 0.1707 | 66.6778 |
| *MaSCL32-1* | *Ma06_t09430.1* | *MaSCL32-4* | *Ma10_t11650.1* | 0.0860 | 0.4938 | 0.1741 | 54.8667 |
| *MaSCL32-2* | *Ma06_t18350.1* | *MaSCL32-4* | *Ma10_t11650.1* | 0.0839 | 0.3540 | 0.2370 | 39.3333 |
| *MaSCL23-2* | *Ma06_t19260.1* | *MaSCL23-3* | *Ma10_t10710.1* | 0.1458 | 0.7317 | 0.1992 | 81.3000 |
| *MaSCL1-2* | *Ma06_t13300.1* | *MaSCL1-3* | *Ma10_t17350.1* | 0.0835 | 0.4475 | 0.1866 | 49.7222 |
| *MaSCL9-2* | *Ma07_t15010.1* | *MaSCL9-4* | *Ma10_t22580.1* | 0.0877 | 0.4528 | 0.1937 | 50.3111 |
| *MaSCL9-2* | *Ma07_t15010.1* | *MaSCL9-4* | *Ma10_t02270.1* | 0.1013 | 0.6204 | 0.1633 | 68.9333 |
| *MaSLR1-3* | *Ma08_t17700.1* | *MaSLN1* | *Ma10_t19630.1* | 0.1032 | 0.4506 | 0.2291 | 50.0667 |
| *MaSCL3-1* | *Ma08_t09470.1* | *MaSCL3-3b* | *Ma11_t18690.2* | 0.1175 | 0.5915 | 0.1987 | 65.7222 |
| *MaSCL27-2* | *Ma08_t10950.1* | *MaSCL27-4* | *Ma11_t19440.1* | 0.1616 | 0.4868 | 0.3321 | 54.0889 |
| *MaSCL27-2* | *Ma08_t10950.1* | *MaSCL27-3* | *Ma11_t04080.1* | 0.0975 | 0.4320 | 0.2256 | 48.0000 |
| *MaCIGR1-3* | *Ma09_t11650.1* | *MaCIGR1-4* | *Ma09_t25150.1* | 0.1063 | 0.8185 | 0.1298 | 90.9444 |
| *MaCIGR1-3* | *Ma09_t02320.1* | *MaSHR2-2* | *Ma09_t20690.1* | 0.0949 | 0.6279 | 0.1511 | 69.7667 |
| *MaCIGR1-3* | *Ma10_t02270.1* | *MaSCL9-4* | *Ma10_t22580.1* | 0.1124 | 0.5578 | 0.2015 | 61.9778 |
| *MaSCL27-3* | *Ma11_t04080.1* | *MaSCL27-4* | *Ma11_t19440.1* | 0.1516 | 0.4916 | 0.3084 | 54.6222 |
| *MbSLR1-1* | *Mba01_g24230.1* | *MbSLR1-2* | *Mba02_g23160.1* | 0.1659 | 0.6331 | 0.2620 | 70.3426 |
| *MbSCL18-1* | *Mba01_g22320.1* | *MbSCL18-3* | *Mba03_g22670.1* | 0.2131 | 0.8373 | 0.2546 | 93.0279 |
| *MbSCL18-1* | *Mba01_g22320.1* | *MbSCL18-2* | *Mba03_g11270.1* | 0.1053 | 0.4505 | 0.2338 | 50.0575 |
| *MbSCL4-1* | *Mba01_g01860.1* | *MbSCL4-2* | *Mba04_g06990.1* | 0.1323 | 0.4955 | 0.2669 | 55.0559 |
| *MbSCL4-1* | *Mba01_g01860.1* | *MbSCL4-4* | *Mba07_g26490.1* | 0.1674 | 0.3926 | 0.4264 | 43.6214 |
| *MbSLR1-1* | *Mba01_g24230.1* | *MbSLN1* | *Mba10_g17320.1* | 0.1559 | 0.7120 | 0.2189 | 79.1081 |
| *MbSLR1-2* | *Mba02_g23160.1* | *MbSLN1* | *Mba10_g17320.1* | 0.0931 | 0.5096 | 0.1827 | 56.6178 |
| *MbSCL1-1* | *Mba03_g02150.1* | *MbSCL1-2* | *Mba06_g12400.1* | 0.1104 | 0.5830 | 0.1893 | 64.7738 |
| *MbSCL18-3* | *Mba03_g22670.1* | *MbSCL18-4* | *Mba07_g16060.1* | 0.1131 | 0.4566 | 0.2477 | 50.7360 |
| *MbSCL1-1* | *Mba03_g02150.1* | *MbSCL1-3* | *Mba10_g15260.1* | 0.1057 | 0.5691 | 0.1858 | 63.2290 |
| *MbSCL8-1* | *Mba04_g24120.1* | *MbSCL8-2* | *Mba04_g32320.1* | 0.1439 | 0.4843 | 0.2970 | 53.8163 |
| *MbSCL8-3* | *Mba04_g33600.1* | *MbSCL8-5* | *Mba05_g15340.1* | 0.1399 | 0.4824 | 0.2900 | 53.6037 |
| *MbSCL8-3* | *Mba04_g33600.1* | *MbSCL8-4* | *Mba05_g05110.1* | 0.1398 | 0.4278 | 0.3269 | 47.5352 |
| *MbSCL6-1* | *Mba04_g26250.1* | *MbSCL6-2* | *Mba06_g37370.1* | 0.1677 | 0.5394 | 0.3110 | 59.9296 |
| *MbSCL6-1* | *Mba04_g26250.1* | *MbSCL6-3* | *Mba07_g06350.1* | 0.1470 | 0.3896 | 0.3773 | 43.2884 |
| *MbSCL8-1* | *Mba04_g24120.1* | *MbSCL8-6* | *Mba09_g18250.1* | 0.1778 | 0.6072 | 0.2928 | 67.4619 |
| *MbSCL8-1* | *Mba04_g24120.1* | *MbSCL8-7* | *Mba11_g02550.1* | 0.2148 | 0.7430 | 0.2892 | 82.5523 |
| *MbSCL8-3* | *Mba04_g33600.1* | *MbSCL8-7* | *Mba11_g02550.1* | 0.2368 | 0.7813 | 0.3031 | 86.8074 |
| *MbSCL8-4* | *Mba05_g05110.1* | *MbSCL8-5* | *Mba05_g15340.1* | 0.1800 | 0.5171 | 0.3481 | 57.4510 |
| *MbSCL27-1* | *Mba05_g22880.1* | *MbSCL27-2* | *Mba08_g10500.1* | 0.1477 | 0.4783 | 0.3089 | 53.1414 |
| *MbSCL27-1* | *Mba05_g22880.1* | *MbSCL27-3* | *Mba11_g04000.1* | 0.1362 | 0.4572 | 0.2978 | 50.8022 |
| *MbSCL8-4* | *Mba05_g05110.1* | *MbSCL8-7* | *Mba11_g02550.1* | 0.2108 | 0.6754 | 0.3122 | 75.0439 |
| *MbSCL8-5* | *Mba05_g15340.1* | *MbSCL8-7* | *Mba11_g02550.1* | 0.2364 | 0.7895 | 0.2994 | 87.7261 |
| *MbSCL9-1* | *Mba06_g34910.1* | *MbSCL9-2* | *Mba07_g13640.1* | 0.3162 | 2.4170 | 0.1308 | 268.5561 |
| *MbSHR2-1* | *Mba06_g21600.1* | *MbSHR2-2* | *Mba09_g02280.1* | 0.0933 | 0.6187 | 0.1509 | 68.7448 |
| *MbSCL32-1* | *Mba06_g17340.1* | *MbSCL32-2* | *Mba10_g10170.1* | 0.0660 | 0.3752 | 0.1759 | 41.6936 |
| *MbSCL1-2* | *Mba06_g12400.1* | *MbSCL1-3* | *Mba10_g15260.1* | 0.0838 | 0.4295 | 0.1951 | 47.7277 |
| *MbSCL27-2* | *Mba08_g10500.1* | *MbSCL27-3* | *Mba11_g04000.1* | 0.0938 | 0.4002 | 0.2343 | 44.4639 |
| *MiSCL15-2* | *Mi_g003925* | *MiSCL15-1* | *Mi_g018794* | 0.1358 | 0.5317 | 0.2554 | 59.0828 |
| *MiSCL15-2* | *Mi_g003925* | *MiSCL15-3* | *Mi_g029761* | 0.1935 | 0.4608 | 0.4200 | 51.1986 |
| *MiSCL9-1* | *Mi_g004290* | *MiSCL9-3* | *Mi_g018131* | 0.2950 | 3.2487 | 0.0908 | 360.9675 |
| *MiSCL32-1* | *Mi_g004371* | *MiSCL32-2* | *Mi_g012984* | 0.0890 | 0.4722 | 0.1885 | 52.4626 |
| *MiSCL32-1* | *Mi_g004371* | *MiSCL32-4* | *Mi_g014628* | 0.0886 | 0.4827 | 0.1835 | 53.6354 |
| *MiSCL32-1* | *Mi_g004371* | *MiSCL21* | *Mi_g029776* | 0.7513 |  |  |  |
| *MiSCL28-2* | *Mi_g007400* | *MiSCL28-1* | *Mi_g024689* | 0.0906 | 0.4531 | 0.1999 | 50.3393 |
| *MiSCL18-3* | *Mi_g008072* | *MiSCL18-2* | *Mi_g027450* | 0.1687 | 0.7096 | 0.2377 | 78.8413 |
| *MiSCL27-2* | *Mi_g012925* | *MiSCL27-1* | *Mi_g028399* | 0.1413 | 0.4879 | 0.2897 | 54.2071 |
| *MiSCL1-3* | *Mi_g015188* | *MiSCL1-2* | *Mi_g017201* |  |  |  |  |
| *MiSCL1-3* | *Mi_g015188* | *MiSCL1-1* | *Mi_g021121* |  |  |  |  |
| *MiSCL23-1* | *Mi_g015524* | *MiSCL23-2* | *Mi_g023914* | 0.1520 | 0.7433 | 0.2044 | 82.5911 |
| *MiSCL1-2* | *Mi_g017201* | *MiSCL1-2* | *Mi_g021121* | 0.0986 | 0.5407 | 0.1824 | 60.0833 |
| *MiSCL4-1* | *Mi_g018749* | *MiSCL1-2* | *Mi_g021121* | 0.8027 |  |  |  |
| *MiSCL15-1* | *Mi_g018794* | *MiCIGR2* | *Mi_g025012* | 0.8959 |  |  |  |
| *MiSCL4-1* | *Mi_g018749* | *MiSCL4-3* | *Mi_g029746* | 0.1225 | 0.5337 | 0.2295 | 59.3031 |

**Supplementary Table 3.** Prediction of miRNA target genes in the MaGRAS family

| MiRNA | Target_ID | Target_name | Expectation | Target_start | Target_end | miRNA_aligned_fragment | Target_aligned_fragment | Inhibition |
| --- | --- | --- | --- | --- | --- | --- | --- | --- |
| mac-miR171a-3p.1 | Ma08_t10950.1 | MaSCL27-2 | 0 | 1299 | 1319 | UGAUUGAGCCGCGCCAAUAUC | GAUAUUGGCGCGGCUCAAUCA | Cleavage |
| mac-miR171a-3p.1 | Ma04_t25650.1 | MaSCL6-1 | 0 | 1146 | 1166 | UGAUUGAGCCGCGCCAAUAUC | GAUAUUGGCGCGGCUCAAUCA | Cleavage |
| mac-miR171a-3p.1 | Ma11_t04080.1 | MaSCL27-3 | 0 | 1311 | 1331 | UGAUUGAGCCGCGCCAAUAUC | GAUAUUGGCGCGGCUCAAUCA | Cleavage |
| mac-miR171a-3p.1 | Ma05_t23270.1 | MaSCL27-1 | 0 | 1296 | 1316 | UGAUUGAGCCGCGCCAAUAUC | GAUAUUGGCGCGGCUCAAUCA | Cleavage |
| mac-miR171a-3p.1 | Ma06_t38750.1 | MaSCL22 | 0 | 426 | 446 | UGAUUGAGCCGCGCCAAUAUC | GAUAUUGGCGCGGCUCAAUCA | Cleavage |
| mac-miR171a-3p.1 | Ma07_t06770.1 | MaSCL6-2 | 0 | 1122 | 1142 | UGAUUGAGCCGCGCCAAUAUC | GAUAUUGGCGCGGCUCAAUCA | Cleavage |
| mac-miR171a-3p.2 | Ma11_t04080.1 | MaSCL27-3 | 0 | 1312 | 1331 | UGAUUGAGCCGCGCCAAUAU | AUAUUGGCGCGGCUCAAUCA | Cleavage |
| mac-miR171a-3p.2 | Ma04_t25650.1 | MaSCL6-1 | 0 | 1147 | 1166 | UGAUUGAGCCGCGCCAAUAU | AUAUUGGCGCGGCUCAAUCA | Cleavage |
| mac-miR171a-3p.2 | Ma05_t23270.1 | MaSCL27-1 | 0 | 1297 | 1316 | UGAUUGAGCCGCGCCAAUAU | AUAUUGGCGCGGCUCAAUCA | Cleavage |
| mac-miR171a-3p.2 | Ma06_t38750.1 | MaSCL22 | 0 | 427 | 446 | UGAUUGAGCCGCGCCAAUAU | AUAUUGGCGCGGCUCAAUCA | Cleavage |
| mac-miR171a-3p.2 | Ma08_t10950.1 | MaSCL27-2 | 0 | 1300 | 1319 | UGAUUGAGCCGCGCCAAUAU | AUAUUGGCGCGGCUCAAUCA | Cleavage |
| mac-miR171a-3p.2 | Ma07_t06770.1 | MaSCL6-2 | 0 | 1123 | 1142 | UGAUUGAGCCGCGCCAAUAU | AUAUUGGCGCGGCUCAAUCA | Cleavage |
| mac-miR171a.2 | Ma11_t19440.1 | MaSCL27-4 | 0 | 1315 | 1334 | UGAUUGAGCCGUGCCAAUAU | AUAUUGGCACGGCUCAAUCA | Cleavage |
| mac-miR171b.2 | Ma11_t19440.1 | MaSCL27-4 | 0 | 1314 | 1334 | UGAUUGAGCCGUGCCAAUAUC | GAUAUUGGCACGGCUCAAUCA | Cleavage |
| mac-miR171c.1 | Ma11_t19440.1 | MaSCL27-4 | 0 | 1314 | 1334 | UGAUUGAGCCGUGCCAAUAUU | GAUAUUGGCACGGCUCAAUCA | Cleavage |
| mac-miR171i | Ma08_t16560.1 | MaNSP2 | 0 | 395 | 415 | UGAGCCGAACCAAUAUCACUC | GGGUGAUAUUGGUUCGGCUCA | Cleavage |
| mac-miR171a.2 | Ma06_t38750.1 | MaSCL22 | 0.5 | 427 | 446 | UGAUUGAGCCGUGCCAAUAU | AUAUUGGCGCGGCUCAAUCA | Cleavage |
| mac-miR171a.2 | Ma04_t25650.1 | MaSCL6-1 | 0.5 | 1147 | 1166 | UGAUUGAGCCGUGCCAAUAU | AUAUUGGCGCGGCUCAAUCA | Cleavage |
| mac-miR171a.2 | Ma11_t04080.1 | MaSCL27-3 | 0.5 | 1312 | 1331 | UGAUUGAGCCGUGCCAAUAU | AUAUUGGCGCGGCUCAAUCA | Cleavage |
| mac-miR171a.2 | Ma05_t23270.1 | MaSCL27-1 | 0.5 | 1297 | 1316 | UGAUUGAGCCGUGCCAAUAU | AUAUUGGCGCGGCUCAAUCA | Cleavage |
| mac-miR171a.2 | Ma07_t06770.1 | MaSCL6-2 | 0.5 | 1123 | 1142 | UGAUUGAGCCGUGCCAAUAU | AUAUUGGCGCGGCUCAAUCA | Cleavage |
| mac-miR171a.2 | Ma08_t10950.1 | MaSCL27-2 | 0.5 | 1300 | 1319 | UGAUUGAGCCGUGCCAAUAU | AUAUUGGCGCGGCUCAAUCA | Cleavage |
| mac-miR171b.1 | Ma08_t10950.1 | MaSCL27-2 | 0.5 | 1299 | 1319 | UGAUUGAGCCGCGUCAAUAUC | GAUAUUGGCGCGGCUCAAUCA | Cleavage |
| mac-miR171b.1 | Ma04_t25650.1 | MaSCL6-1 | 0.5 | 1146 | 1166 | UGAUUGAGCCGCGUCAAUAUC | GAUAUUGGCGCGGCUCAAUCA | Cleavage |
| mac-miR171b.1 | Ma11_t04080.1 | MaSCL27-3 | 0.5 | 1311 | 1331 | UGAUUGAGCCGCGUCAAUAUC | GAUAUUGGCGCGGCUCAAUCA | Cleavage |
| mac-miR171b.1 | Ma05_t23270.1 | MaSCL27-1 | 0.5 | 1296 | 1316 | UGAUUGAGCCGCGUCAAUAUC | GAUAUUGGCGCGGCUCAAUCA | Cleavage |
| mac-miR171b.1 | Ma06_t38750.1 | MaSCL22 | 0.5 | 426 | 446 | UGAUUGAGCCGCGUCAAUAUC | GAUAUUGGCGCGGCUCAAUCA | Cleavage |
| mac-miR171b.1 | Ma07_t06770.1 | MaSCL6-2 | 0.5 | 1122 | 1142 | UGAUUGAGCCGCGUCAAUAUC | GAUAUUGGCGCGGCUCAAUCA | Cleavage |
| mac-miR171b.2 | Ma07_t06770.1 | MaSCL6-2 | 0.5 | 1122 | 1142 | UGAUUGAGCCGUGCCAAUAUC | GAUAUUGGCGCGGCUCAAUCA | Cleavage |
| mac-miR171b.2 | Ma04_t25650.1 | MaSCL6-1 | 0.5 | 1146 | 1166 | UGAUUGAGCCGUGCCAAUAUC | GAUAUUGGCGCGGCUCAAUCA | Cleavage |
| mac-miR171b.2 | Ma08_t10950.1 | MaSCL27-2 | 0.5 | 1299 | 1319 | UGAUUGAGCCGUGCCAAUAUC | GAUAUUGGCGCGGCUCAAUCA | Cleavage |
| mac-miR171b.2 | Ma11_t04080.1 | MaSCL27-3 | 0.5 | 1311 | 1331 | UGAUUGAGCCGUGCCAAUAUC | GAUAUUGGCGCGGCUCAAUCA | Cleavage |
| mac-miR171b.2 | Ma05_t23270.1 | MaSCL27-1 | 0.5 | 1296 | 1316 | UGAUUGAGCCGUGCCAAUAUC | GAUAUUGGCGCGGCUCAAUCA | Cleavage |
| mac-miR171b.2 | Ma06_t38750.1 | MaSCL22 | 0.5 | 426 | 446 | UGAUUGAGCCGUGCCAAUAUC | GAUAUUGGCGCGGCUCAAUCA | Cleavage |
| mac-miR171c.1 | Ma07_t06770.1 | MaSCL6-2 | 0.5 | 1122 | 1142 | UGAUUGAGCCGUGCCAAUAUU | GAUAUUGGCGCGGCUCAAUCA | Cleavage |
| mac-miR171c.1 | Ma04_t25650.1 | MaSCL6-1 | 0.5 | 1146 | 1166 | UGAUUGAGCCGUGCCAAUAUU | GAUAUUGGCGCGGCUCAAUCA | Cleavage |
| mac-miR171c.1 | Ma11_t04080.1 | MaSCL27-3 | 0.5 | 1311 | 1331 | UGAUUGAGCCGUGCCAAUAUU | GAUAUUGGCGCGGCUCAAUCA | Cleavage |
| mac-miR171c.1 | Ma08_t10950.1 | MaSCL27-2 | 0.5 | 1299 | 1319 | UGAUUGAGCCGUGCCAAUAUU | GAUAUUGGCGCGGCUCAAUCA | Cleavage |
| mac-miR171c.1 | Ma06_t38750.1 | MaSCL22 | 0.5 | 426 | 446 | UGAUUGAGCCGUGCCAAUAUU | GAUAUUGGCGCGGCUCAAUCA | Cleavage |
| mac-miR171c.1 | Ma05_t23270.1 | MaSCL27-1 | 0.5 | 1296 | 1316 | UGAUUGAGCCGUGCCAAUAUU | GAUAUUGGCGCGGCUCAAUCA | Cleavage |
| mac-miR171h.2 | Ma07_t06770.1 | MaSCL6-2 | 0.5 | 1122 | 1142 | UGGUUGAGCCGCGCCAAUAUC | GAUAUUGGCGCGGCUCAAUCA | Cleavage |
| mac-miR171h.2 | Ma08_t10950.1 | MaSCL27-2 | 0.5 | 1299 | 1319 | UGGUUGAGCCGCGCCAAUAUC | GAUAUUGGCGCGGCUCAAUCA | Cleavage |
| mac-miR171h.2 | Ma04_t25650.1 | MaSCL6-1 | 0.5 | 1146 | 1166 | UGGUUGAGCCGCGCCAAUAUC | GAUAUUGGCGCGGCUCAAUCA | Cleavage |
| mac-miR171h.2 | Ma06_t38750.1 | MaSCL22 | 0.5 | 426 | 446 | UGGUUGAGCCGCGCCAAUAUC | GAUAUUGGCGCGGCUCAAUCA | Cleavage |
| mac-miR171h.2 | Ma11_t04080.1 | MaSCL27-3 | 0.5 | 1311 | 1331 | UGGUUGAGCCGCGCCAAUAUC | GAUAUUGGCGCGGCUCAAUCA | Cleavage |
| mac-miR171h.2 | Ma05_t23270.1 | MaSCL27-1 | 0.5 | 1296 | 1316 | UGGUUGAGCCGCGCCAAUAUC | GAUAUUGGCGCGGCUCAAUCA | Cleavage |
| mac-miR171 | Ma06_t38750.1 | MaSCL22 | 1 | 423 | 443 | UUGAGCCGCGUCAAUAUCUCC | UGGGAUAUUGGCGCGGCUCAA | Cleavage |
| mac-miR171 | Ma05_t23270.1 | MaSCL27-1 | 1 | 1293 | 1313 | UUGAGCCGCGUCAAUAUCUCC | CGGGAUAUUGGCGCGGCUCAA | Cleavage |
| mac-miR171 | Ma07_t06770.1 | MaSCL6-2 | 1 | 1119 | 1139 | UUGAGCCGCGUCAAUAUCUCC | AGGGAUAUUGGCGCGGCUCAA | Cleavage |
| mac-miR171 | Ma08_t10950.1 | MaSCL27-2 | 1 | 1296 | 1316 | UUGAGCCGCGUCAAUAUCUCC | UGGGAUAUUGGCGCGGCUCAA | Cleavage |
| mac-miR171 | Ma04_t25650.1 | MaSCL6-1 | 1 | 1143 | 1163 | UUGAGCCGCGUCAAUAUCUCC | UGGGAUAUUGGCGCGGCUCAA | Cleavage |
| mac-miR171a-3p.1 | Ma04_t03560.1 | MaSCL15-2 | 1 | 543 | 563 | UGAUUGAGCCGCGCCAAUAUC | GAUAUUGUCGCGGCUCAAUCA | Cleavage |
| mac-miR171a-3p.1 | Ma03_t31190.1 | MaSCL15-1 | 1 | 543 | 563 | UGAUUGAGCCGCGCCAAUAUC | GAUAUUGUCGCGGCUCAAUCA | Cleavage |
| mac-miR171a-3p.1 | Ma04_t07170.1 | MaSCL15-3 | 1 | 504 | 524 | UGAUUGAGCCGCGCCAAUAUC | GAUAUUGUCGCGGCUCAAUCA | Cleavage |
| mac-miR171a-3p.2 | Ma03_t31190.1 | MaSCL15-1 | 1 | 544 | 563 | UGAUUGAGCCGCGCCAAUAU | AUAUUGUCGCGGCUCAAUCA | Cleavage |
| mac-miR171a-3p.2 | Ma04_t07170.1 | MaSCL15-3 | 1 | 505 | 524 | UGAUUGAGCCGCGCCAAUAU | AUAUUGUCGCGGCUCAAUCA | Cleavage |
| mac-miR171a-3p.2 | Ma04_t03560.1 | MaSCL15-2 | 1 | 544 | 563 | UGAUUGAGCCGCGCCAAUAU | AUAUUGUCGCGGCUCAAUCA | Cleavage |
| mac-miR171a.1 | Ma11_t19440.1 | MaSCL27-4 | 1 | 1310 | 1330 | UGAGCCGUGCCAAUAUCACGA | AUGGGAUAUUGGCACGGCUCA | Cleavage |
| mac-miR171a.3 | Ma08_t10950.1 | MaSCL27-2 | 1 | 1295 | 1315 | UGAGCCGCGCCAAUAUCACAU | AUGGGAUAUUGGCGCGGCUCA | Cleavage |
| mac-miR171a.3 | Ma06_t38750.1 | MaSCL22 | 1 | 422 | 442 | UGAGCCGCGCCAAUAUCACAU | GUGGGAUAUUGGCGCGGCUCA | Cleavage |
| mac-miR171a.3 | Ma04_t25650.1 | MaSCL6-1 | 1 | 1142 | 1162 | UGAGCCGCGCCAAUAUCACAU | GUGGGAUAUUGGCGCGGCUCA | Cleavage |
| mac-miR171a.3 | Ma11_t04080.1 | MaSCL27-3 | 1 | 1307 | 1327 | UGAGCCGCGCCAAUAUCACAU | ACGCGAUAUUGGCGCGGCUCA | Cleavage |
| mac-miR171a.3 | Ma05_t23270.1 | MaSCL27-1 | 1 | 1292 | 1312 | UGAGCCGCGCCAAUAUCACAU | ACGGGAUAUUGGCGCGGCUCA | Cleavage |
| mac-miR171a.3 | Ma07_t06770.1 | MaSCL6-2 | 1 | 1118 | 1138 | UGAGCCGCGCCAAUAUCACAU | GAGGGAUAUUGGCGCGGCUCA | Cleavage |
| mac-miR171b-3p.1 | Ma11_t19440.1 | MaSCL27-4 | 1 | 1311 | 1331 | UUGAGCCGUGCCAAUAUCACG | UGGGAUAUUGGCACGGCUCAA | Cleavage |
| mac-miR171b-3p.2 | Ma07_t06770.1 | MaSCL6-2 | 1 | 1119 | 1139 | UUGAGCCGCGUCAAUAUCUCU | AGGGAUAUUGGCGCGGCUCAA | Cleavage |
| mac-miR171b-3p.2 | Ma04_t25650.1 | MaSCL6-1 | 1 | 1143 | 1163 | UUGAGCCGCGUCAAUAUCUCU | UGGGAUAUUGGCGCGGCUCAA | Cleavage |
| mac-miR171b-3p.2 | Ma05_t23270.1 | MaSCL27-1 | 1 | 1293 | 1313 | UUGAGCCGCGUCAAUAUCUCU | CGGGAUAUUGGCGCGGCUCAA | Cleavage |
| mac-miR171b-3p.2 | Ma08_t10950.1 | MaSCL27-2 | 1 | 1296 | 1316 | UUGAGCCGCGUCAAUAUCUCU | UGGGAUAUUGGCGCGGCUCAA | Cleavage |
| mac-miR171b-3p.2 | Ma06_t38750.1 | MaSCL22 | 1 | 423 | 443 | UUGAGCCGCGUCAAUAUCUCU | UGGGAUAUUGGCGCGGCUCAA | Cleavage |
| mac-miR171b-3p.3 | Ma11_t19440.1 | MaSCL27-4 | 1 | 1312 | 1331 | UUGAGCCGUGCCAAUAUCAC | GGGAUAUUGGCACGGCUCAA | Cleavage |
| mac-miR171b.1 | Ma04_t03560.1 | MaSCL15-2 | 1 | 543 | 563 | UGAUUGAGCCGCGUCAAUAUC | GAUAUUGUCGCGGCUCAAUCA | Cleavage |
| mac-miR171b.1 | Ma03_t31190.1 | MaSCL15-1 | 1 | 543 | 563 | UGAUUGAGCCGCGUCAAUAUC | GAUAUUGUCGCGGCUCAAUCA | Cleavage |
| mac-miR171b.1 | Ma04_t07170.1 | MaSCL15-3 | 1 | 504 | 524 | UGAUUGAGCCGCGUCAAUAUC | GAUAUUGUCGCGGCUCAAUCA | Cleavage |
| mac-miR171b.3 | Ma08_t10950.1 | MaSCL27-2 | 1 | 1296 | 1316 | UUGAGCCGCGCCAAUAUCACA | UGGGAUAUUGGCGCGGCUCAA | Cleavage |
| mac-miR171b.3 | Ma04_t25650.1 | MaSCL6-1 | 1 | 1143 | 1163 | UUGAGCCGCGCCAAUAUCACA | UGGGAUAUUGGCGCGGCUCAA | Cleavage |
| mac-miR171b.3 | Ma06_t38750.1 | MaSCL22 | 1 | 423 | 443 | UUGAGCCGCGCCAAUAUCACA | UGGGAUAUUGGCGCGGCUCAA | Cleavage |
| mac-miR171b.3 | Ma11_t04080.1 | MaSCL27-3 | 1 | 1308 | 1328 | UUGAGCCGCGCCAAUAUCACA | CGCGAUAUUGGCGCGGCUCAA | Cleavage |
| mac-miR171b.3 | Ma05_t23270.1 | MaSCL27-1 | 1 | 1293 | 1313 | UUGAGCCGCGCCAAUAUCACA | CGGGAUAUUGGCGCGGCUCAA | Cleavage |
| mac-miR171b.3 | Ma07_t06770.1 | MaSCL6-2 | 1 | 1119 | 1139 | UUGAGCCGCGCCAAUAUCACA | AGGGAUAUUGGCGCGGCUCAA | Cleavage |
| mac-miR171c.2 | Ma11_t04080.1 | MaSCL27-3 | 1 | 1311 | 1331 | AGAUUGAGCCGCGCCAAUAUC | GAUAUUGGCGCGGCUCAAUCA | Cleavage |
| mac-miR171c.2 | Ma05_t23270.1 | MaSCL27-1 | 1 | 1296 | 1316 | AGAUUGAGCCGCGCCAAUAUC | GAUAUUGGCGCGGCUCAAUCA | Cleavage |
| mac-miR171c.2 | Ma07_t06770.1 | MaSCL6-2 | 1 | 1122 | 1142 | AGAUUGAGCCGCGCCAAUAUC | GAUAUUGGCGCGGCUCAAUCA | Cleavage |
| mac-miR171c.2 | Ma08_t10950.1 | MaSCL27-2 | 1 | 1299 | 1319 | AGAUUGAGCCGCGCCAAUAUC | GAUAUUGGCGCGGCUCAAUCA | Cleavage |
| mac-miR171c.2 | Ma04_t25650.1 | MaSCL6-1 | 1 | 1146 | 1166 | AGAUUGAGCCGCGCCAAUAUC | GAUAUUGGCGCGGCUCAAUCA | Cleavage |
| mac-miR171c.2 | Ma06_t38750.1 | MaSCL22 | 1 | 426 | 446 | AGAUUGAGCCGCGCCAAUAUC | GAUAUUGGCGCGGCUCAAUCA | Cleavage |
| mac-miR171d | Ma06_t38750.1 | MaSCL22 | 1 | 424 | 443 | UUGAGCCGCGCCAAUAUCAC | GGGAUAUUGGCGCGGCUCAA | Cleavage |
| mac-miR171d | Ma08_t10950.1 | MaSCL27-2 | 1 | 1297 | 1316 | UUGAGCCGCGCCAAUAUCAC | GGGAUAUUGGCGCGGCUCAA | Cleavage |
| mac-miR171d | Ma11_t04080.1 | MaSCL27-3 | 1 | 1309 | 1328 | UUGAGCCGCGCCAAUAUCAC | GCGAUAUUGGCGCGGCUCAA | Cleavage |
| mac-miR171d | Ma05_t23270.1 | MaSCL27-1 | 1 | 1294 | 1313 | UUGAGCCGCGCCAAUAUCAC | GGGAUAUUGGCGCGGCUCAA | Cleavage |
| mac-miR171d | Ma07_t06770.1 | MaSCL6-2 | 1 | 1120 | 1139 | UUGAGCCGCGCCAAUAUCAC | GGGAUAUUGGCGCGGCUCAA | Cleavage |
| mac-miR171d | Ma04_t25650.1 | MaSCL6-1 | 1 | 1144 | 1163 | UUGAGCCGCGCCAAUAUCAC | GGGAUAUUGGCGCGGCUCAA | Cleavage |
| mac-miR171f.1 | Ma08_t16560.1 | MaNSP2 | 1 | 396 | 416 | AUGAGCCGAACCAAUAUCACU | GGUGAUAUUGGUUCGGCUCAA | Cleavage |
| mac-miR171f.2 | Ma07_t06770.1 | MaSCL6-2 | 1 | 1119 | 1139 | UUGAGCCGCGCCAAUAUCACU | AGGGAUAUUGGCGCGGCUCAA | Cleavage |
| mac-miR171f.2 | Ma11_t04080.1 | MaSCL27-3 | 1 | 1308 | 1328 | UUGAGCCGCGCCAAUAUCACU | CGCGAUAUUGGCGCGGCUCAA | Cleavage |
| mac-miR171f.2 | Ma05_t23270.1 | MaSCL27-1 | 1 | 1293 | 1313 | UUGAGCCGCGCCAAUAUCACU | CGGGAUAUUGGCGCGGCUCAA | Cleavage |
| mac-miR171f.2 | Ma08_t10950.1 | MaSCL27-2 | 1 | 1296 | 1316 | UUGAGCCGCGCCAAUAUCACU | UGGGAUAUUGGCGCGGCUCAA | Cleavage |
| mac-miR171f.2 | Ma06_t38750.1 | MaSCL22 | 1 | 423 | 443 | UUGAGCCGCGCCAAUAUCACU | UGGGAUAUUGGCGCGGCUCAA | Cleavage |
| mac-miR171f.2 | Ma04_t25650.1 | MaSCL6-1 | 1 | 1143 | 1163 | UUGAGCCGCGCCAAUAUCACU | UGGGAUAUUGGCGCGGCUCAA | Cleavage |
| mac-miR171h.1 | Ma08_t16560.1 | MaNSP2 | 1 | 396 | 416 | GUGAGCCGAACCAAUAUCACU | GGUGAUAUUGGUUCGGCUCAA | Cleavage |
| mac-miR171k | Ma05_t23270.1 | MaSCL27-1 | 1 | 1296 | 1316 | GGAUUGAGCCGCGCCAAUAUC | GAUAUUGGCGCGGCUCAAUCA | Cleavage |
| mac-miR171k | Ma08_t10950.1 | MaSCL27-2 | 1 | 1299 | 1319 | GGAUUGAGCCGCGCCAAUAUC | GAUAUUGGCGCGGCUCAAUCA | Cleavage |
| mac-miR171k | Ma11_t04080.1 | MaSCL27-3 | 1 | 1311 | 1331 | GGAUUGAGCCGCGCCAAUAUC | GAUAUUGGCGCGGCUCAAUCA | Cleavage |
| mac-miR171k | Ma07_t06770.1 | MaSCL6-2 | 1 | 1122 | 1142 | GGAUUGAGCCGCGCCAAUAUC | GAUAUUGGCGCGGCUCAAUCA | Cleavage |
| mac-miR171k | Ma04_t25650.1 | MaSCL6-1 | 1 | 1146 | 1166 | GGAUUGAGCCGCGCCAAUAUC | GAUAUUGGCGCGGCUCAAUCA | Cleavage |
| mac-miR171k | Ma06_t38750.1 | MaSCL22 | 1 | 426 | 446 | GGAUUGAGCCGCGCCAAUAUC | GAUAUUGGCGCGGCUCAAUCA | Cleavage |
| mac-miR171m | Ma06_t38750.1 | MaSCL22 | 1 | 423 | 443 | UUGAGCCGCGUCAAUAUCUCA | UGGGAUAUUGGCGCGGCUCAA | Cleavage |
| mac-miR171m | Ma04_t25650.1 | MaSCL6-1 | 1 | 1143 | 1163 | UUGAGCCGCGUCAAUAUCUCA | UGGGAUAUUGGCGCGGCUCAA | Cleavage |
| mac-miR171m | Ma08_t10950.1 | MaSCL27-2 | 1 | 1296 | 1316 | UUGAGCCGCGUCAAUAUCUCA | UGGGAUAUUGGCGCGGCUCAA | Cleavage |
| mac-miR171m | Ma07_t06770.1 | MaSCL6-2 | 1 | 1119 | 1139 | UUGAGCCGCGUCAAUAUCUCA | AGGGAUAUUGGCGCGGCUCAA | Cleavage |
| mac-miR171m | Ma05_t23270.1 | MaSCL27-1 | 1 | 1293 | 1313 | UUGAGCCGCGUCAAUAUCUCA | CGGGAUAUUGGCGCGGCUCAA | Cleavage |
| mac-miR171n | Ma08_t10950.1 | MaSCL27-2 | 1 | 1296 | 1316 | UUGAGCCGCGUCAAUAUCUUA | UGGGAUAUUGGCGCGGCUCAA | Cleavage |
| mac-miR171n | Ma04_t25650.1 | MaSCL6-1 | 1 | 1143 | 1163 | UUGAGCCGCGUCAAUAUCUUA | UGGGAUAUUGGCGCGGCUCAA | Cleavage |
| mac-miR171n | Ma06_t38750.1 | MaSCL22 | 1 | 423 | 443 | UUGAGCCGCGUCAAUAUCUUA | UGGGAUAUUGGCGCGGCUCAA | Cleavage |
| mac-miR171n | Ma07_t06770.1 | MaSCL6-2 | 1 | 1119 | 1139 | UUGAGCCGCGUCAAUAUCUUA | AGGGAUAUUGGCGCGGCUCAA | Cleavage |
| mac-miR171n | Ma05_t23270.1 | MaSCL27-1 | 1 | 1293 | 1313 | UUGAGCCGCGUCAAUAUCUUA | CGGGAUAUUGGCGCGGCUCAA | Cleavage |
| mac-miR171 | Ma11_t04080.1 | MaSCL27-3 | 1.5 | 1308 | 1328 | UUGAGCCGCGUCAAUAUCUCC | CGCGAUAUUGGCGCGGCUCAA | Cleavage |
| mac-miR171a-3p.1 | Ma11_t19440.1 | MaSCL27-4 | 1.5 | 1314 | 1334 | UGAUUGAGCCGCGCCAAUAUC | GAUAUUGGCACGGCUCAAUCA | Cleavage |
| mac-miR171a-3p.2 | Ma11_t19440.1 | MaSCL27-4 | 1.5 | 1315 | 1334 | UGAUUGAGCCGCGCCAAUAU | AUAUUGGCACGGCUCAAUCA | Cleavage |
| mac-miR171a.1 | Ma11_t04080.1 | MaSCL27-3 | 1.5 | 1307 | 1327 | UGAGCCGUGCCAAUAUCACGA | ACGCGAUAUUGGCGCGGCUCA | Cleavage |
| mac-miR171a.1 | Ma05_t23270.1 | MaSCL27-1 | 1.5 | 1292 | 1312 | UGAGCCGUGCCAAUAUCACGA | ACGGGAUAUUGGCGCGGCUCA | Cleavage |
| mac-miR171a.1 | Ma08_t10950.1 | MaSCL27-2 | 1.5 | 1295 | 1315 | UGAGCCGUGCCAAUAUCACGA | AUGGGAUAUUGGCGCGGCUCA | Cleavage |
| mac-miR171a.1 | Ma06_t38750.1 | MaSCL22 | 1.5 | 422 | 442 | UGAGCCGUGCCAAUAUCACGA | GUGGGAUAUUGGCGCGGCUCA | Cleavage |
| mac-miR171a.1 | Ma04_t25650.1 | MaSCL6-1 | 1.5 | 1142 | 1162 | UGAGCCGUGCCAAUAUCACGA | GUGGGAUAUUGGCGCGGCUCA | Cleavage |
| mac-miR171a.1 | Ma07_t06770.1 | MaSCL6-2 | 1.5 | 1118 | 1138 | UGAGCCGUGCCAAUAUCACGA | GAGGGAUAUUGGCGCGGCUCA | Cleavage |
| mac-miR171a.2 | Ma03_t31190.1 | MaSCL15-1 | 1.5 | 544 | 563 | UGAUUGAGCCGUGCCAAUAU | AUAUUGUCGCGGCUCAAUCA | Cleavage |
| mac-miR171a.2 | Ma04_t07170.1 | MaSCL15-3 | 1.5 | 505 | 524 | UGAUUGAGCCGUGCCAAUAU | AUAUUGUCGCGGCUCAAUCA | Cleavage |
| mac-miR171a.2 | Ma04_t03560.1 | MaSCL15-2 | 1.5 | 544 | 563 | UGAUUGAGCCGUGCCAAUAU | AUAUUGUCGCGGCUCAAUCA | Cleavage |
| mac-miR171a.3 | Ma03_t31190.1 | MaSCL15-1 | 1.5 | 539 | 559 | UGAGCCGCGCCAAUAUCACAU | ACGUGAUAUUGUCGCGGCUCA | Translation |
| mac-miR171a.3 | Ma04_t03560.1 | MaSCL15-2 | 1.5 | 539 | 559 | UGAGCCGCGCCAAUAUCACAU | ACGUGAUAUUGUCGCGGCUCA | Translation |
| mac-miR171a.3 | Ma04_t07170.1 | MaSCL15-3 | 1.5 | 500 | 520 | UGAGCCGCGCCAAUAUCACAU | GCGUGAUAUUGUCGCGGCUCA | Translation |
| mac-miR171b-3p.1 | Ma11_t04080.1 | MaSCL27-3 | 1.5 | 1308 | 1328 | UUGAGCCGUGCCAAUAUCACG | CGCGAUAUUGGCGCGGCUCAA | Cleavage |
| mac-miR171b-3p.1 | Ma05_t23270.1 | MaSCL27-1 | 1.5 | 1293 | 1313 | UUGAGCCGUGCCAAUAUCACG | CGGGAUAUUGGCGCGGCUCAA | Cleavage |
| mac-miR171b-3p.1 | Ma04_t25650.1 | MaSCL6-1 | 1.5 | 1143 | 1163 | UUGAGCCGUGCCAAUAUCACG | UGGGAUAUUGGCGCGGCUCAA | Cleavage |
| mac-miR171b-3p.1 | Ma08_t10950.1 | MaSCL27-2 | 1.5 | 1296 | 1316 | UUGAGCCGUGCCAAUAUCACG | UGGGAUAUUGGCGCGGCUCAA | Cleavage |
| mac-miR171b-3p.1 | Ma06_t38750.1 | MaSCL22 | 1.5 | 423 | 443 | UUGAGCCGUGCCAAUAUCACG | UGGGAUAUUGGCGCGGCUCAA | Cleavage |
| mac-miR171b-3p.1 | Ma07_t06770.1 | MaSCL6-2 | 1.5 | 1119 | 1139 | UUGAGCCGUGCCAAUAUCACG | AGGGAUAUUGGCGCGGCUCAA | Cleavage |
| mac-miR171b-3p.2 | Ma11_t04080.1 | MaSCL27-3 | 1.5 | 1308 | 1328 | UUGAGCCGCGUCAAUAUCUCU | CGCGAUAUUGGCGCGGCUCAA | Cleavage |
| mac-miR171b-3p.3 | Ma05_t23270.1 | MaSCL27-1 | 1.5 | 1294 | 1313 | UUGAGCCGUGCCAAUAUCAC | GGGAUAUUGGCGCGGCUCAA | Cleavage |
| mac-miR171b-3p.3 | Ma07_t06770.1 | MaSCL6-2 | 1.5 | 1120 | 1139 | UUGAGCCGUGCCAAUAUCAC | GGGAUAUUGGCGCGGCUCAA | Cleavage |
| mac-miR171b-3p.3 | Ma06_t38750.1 | MaSCL22 | 1.5 | 424 | 443 | UUGAGCCGUGCCAAUAUCAC | GGGAUAUUGGCGCGGCUCAA | Cleavage |
| mac-miR171b-3p.3 | Ma11_t04080.1 | MaSCL27-3 | 1.5 | 1309 | 1328 | UUGAGCCGUGCCAAUAUCAC | GCGAUAUUGGCGCGGCUCAA | Cleavage |
| mac-miR171b-3p.3 | Ma08_t10950.1 | MaSCL27-2 | 1.5 | 1297 | 1316 | UUGAGCCGUGCCAAUAUCAC | GGGAUAUUGGCGCGGCUCAA | Cleavage |
| mac-miR171b-3p.3 | Ma04_t25650.1 | MaSCL6-1 | 1.5 | 1144 | 1163 | UUGAGCCGUGCCAAUAUCAC | GGGAUAUUGGCGCGGCUCAA | Cleavage |
| mac-miR171b.2 | Ma04_t07170.1 | MaSCL15-3 | 1.5 | 504 | 524 | UGAUUGAGCCGUGCCAAUAUC | GAUAUUGUCGCGGCUCAAUCA | Cleavage |
| mac-miR171b.2 | Ma04_t03560.1 | MaSCL15-2 | 1.5 | 543 | 563 | UGAUUGAGCCGUGCCAAUAUC | GAUAUUGUCGCGGCUCAAUCA | Cleavage |
| mac-miR171b.2 | Ma03_t31190.1 | MaSCL15-1 | 1.5 | 543 | 563 | UGAUUGAGCCGUGCCAAUAUC | GAUAUUGUCGCGGCUCAAUCA | Cleavage |
| mac-miR171b.3 | Ma04_t07170.1 | MaSCL15-3 | 1.5 | 501 | 521 | UUGAGCCGCGCCAAUAUCACA | CGUGAUAUUGUCGCGGCUCAA | Translation |
| mac-miR171b.3 | Ma03_t31190.1 | MaSCL15-1 | 1.5 | 540 | 560 | UUGAGCCGCGCCAAUAUCACA | CGUGAUAUUGUCGCGGCUCAA | Translation |
| mac-miR171b.3 | Ma04_t03560.1 | MaSCL15-2 | 1.5 | 540 | 560 | UUGAGCCGCGCCAAUAUCACA | CGUGAUAUUGUCGCGGCUCAA | Translation |
| mac-miR171c.1 | Ma04_t07170.1 | MaSCL15-3 | 1.5 | 504 | 524 | UGAUUGAGCCGUGCCAAUAUU | GAUAUUGUCGCGGCUCAAUCA | Cleavage |
| mac-miR171c.1 | Ma04_t03560.1 | MaSCL15-2 | 1.5 | 543 | 563 | UGAUUGAGCCGUGCCAAUAUU | GAUAUUGUCGCGGCUCAAUCA | Cleavage |
| mac-miR171c.1 | Ma03_t31190.1 | MaSCL15-1 | 1.5 | 543 | 563 | UGAUUGAGCCGUGCCAAUAUU | GAUAUUGUCGCGGCUCAAUCA | Cleavage |
| mac-miR171d | Ma03_t31190.1 | MaSCL15-1 | 1.5 | 541 | 560 | UUGAGCCGCGCCAAUAUCAC | GUGAUAUUGUCGCGGCUCAA | Translation |
| mac-miR171d | Ma04_t03560.1 | MaSCL15-2 | 1.5 | 541 | 560 | UUGAGCCGCGCCAAUAUCAC | GUGAUAUUGUCGCGGCUCAA | Translation |
| mac-miR171d | Ma04_t07170.1 | MaSCL15-3 | 1.5 | 502 | 521 | UUGAGCCGCGCCAAUAUCAC | GUGAUAUUGUCGCGGCUCAA | Translation |
| mac-miR171f | Ma11_t19440.1 | MaSCL27-4 | 1.5 | 1314 | 1334 | UAAUUGAGCCGUGCCAAUAUC | GAUAUUGGCACGGCUCAAUCA | Cleavage |
| mac-miR171f.2 | Ma04_t03560.1 | MaSCL15-2 | 1.5 | 540 | 560 | UUGAGCCGCGCCAAUAUCACU | CGUGAUAUUGUCGCGGCUCAA | Translation |
| mac-miR171f.2 | Ma04_t07170.1 | MaSCL15-3 | 1.5 | 501 | 521 | UUGAGCCGCGCCAAUAUCACU | CGUGAUAUUGUCGCGGCUCAA | Translation |
| mac-miR171f.2 | Ma03_t31190.1 | MaSCL15-1 | 1.5 | 540 | 560 | UUGAGCCGCGCCAAUAUCACU | CGUGAUAUUGUCGCGGCUCAA | Translation |
| mac-miR171h.2 | Ma04_t03560.1 | MaSCL15-2 | 1.5 | 543 | 563 | UGGUUGAGCCGCGCCAAUAUC | GAUAUUGUCGCGGCUCAAUCA | Cleavage |
| mac-miR171h.2 | Ma04_t07170.1 | MaSCL15-3 | 1.5 | 504 | 524 | UGGUUGAGCCGCGCCAAUAUC | GAUAUUGUCGCGGCUCAAUCA | Cleavage |
| mac-miR171h.2 | Ma03_t31190.1 | MaSCL15-1 | 1.5 | 543 | 563 | UGGUUGAGCCGCGCCAAUAUC | GAUAUUGUCGCGGCUCAAUCA | Cleavage |
| mac-miR171i-3p | Ma07_t06770.1 | MaSCL6-2 | 1.5 | 1122 | 1142 | GGAUUGAGCCGCGUCAAUAUC | GAUAUUGGCGCGGCUCAAUCA | Cleavage |
| mac-miR171i-3p | Ma06_t38750.1 | MaSCL22 | 1.5 | 426 | 446 | GGAUUGAGCCGCGUCAAUAUC | GAUAUUGGCGCGGCUCAAUCA | Cleavage |
| mac-miR171i-3p | Ma04_t25650.1 | MaSCL6-1 | 1.5 | 1146 | 1166 | GGAUUGAGCCGCGUCAAUAUC | GAUAUUGGCGCGGCUCAAUCA | Cleavage |
| mac-miR171i-3p | Ma08_t10950.1 | MaSCL27-2 | 1.5 | 1299 | 1319 | GGAUUGAGCCGCGUCAAUAUC | GAUAUUGGCGCGGCUCAAUCA | Cleavage |
| mac-miR171i-3p | Ma11_t04080.1 | MaSCL27-3 | 1.5 | 1311 | 1331 | GGAUUGAGCCGCGUCAAUAUC | GAUAUUGGCGCGGCUCAAUCA | Cleavage |
| mac-miR171i-3p | Ma05_t23270.1 | MaSCL27-1 | 1.5 | 1296 | 1316 | GGAUUGAGCCGCGUCAAUAUC | GAUAUUGGCGCGGCUCAAUCA | Cleavage |
| mac-miR171m | Ma11_t04080.1 | MaSCL27-3 | 1.5 | 1308 | 1328 | UUGAGCCGCGUCAAUAUCUCA | CGCGAUAUUGGCGCGGCUCAA | Cleavage |
| mac-miR171n | Ma11_t04080.1 | MaSCL27-3 | 1.5 | 1308 | 1328 | UUGAGCCGCGUCAAUAUCUUA | CGCGAUAUUGGCGCGGCUCAA | Cleavage |
| mac-miR171a.1 | Ma04_t07170.1 | MaSCL15-3 | 2 | 500 | 520 | UGAGCCGUGCCAAUAUCACGA | GCGUGAUAUUGUCGCGGCUCA | Translation |
| mac-miR171a.1 | Ma04_t03560.1 | MaSCL15-2 | 2 | 539 | 559 | UGAGCCGUGCCAAUAUCACGA | ACGUGAUAUUGUCGCGGCUCA | Translation |
| mac-miR171a.1 | Ma03_t31190.1 | MaSCL15-1 | 2 | 539 | 559 | UGAGCCGUGCCAAUAUCACGA | ACGUGAUAUUGUCGCGGCUCA | Translation |
| mac-miR171a.1 | Ma08_t16560.1 | MaNSP2 | 2 | 395 | 415 | UGAGCCGUGCCAAUAUCACGA | GGGUGAUAUUGGUUCGGCUCA | Cleavage |
| mac-miR171a.3 | Ma08_t16560.1 | MaNSP2 | 2 | 395 | 415 | UGAGCCGCGCCAAUAUCACAU | GGGUGAUAUUGGUUCGGCUCA | Cleavage |
| mac-miR171b-3p.1 | Ma04_t03560.1 | MaSCL15-2 | 2 | 540 | 560 | UUGAGCCGUGCCAAUAUCACG | CGUGAUAUUGUCGCGGCUCAA | Translation |
| mac-miR171b-3p.1 | Ma03_t31190.1 | MaSCL15-1 | 2 | 540 | 560 | UUGAGCCGUGCCAAUAUCACG | CGUGAUAUUGUCGCGGCUCAA | Translation |
| mac-miR171b-3p.1 | Ma04_t07170.1 | MaSCL15-3 | 2 | 501 | 521 | UUGAGCCGUGCCAAUAUCACG | CGUGAUAUUGUCGCGGCUCAA | Translation |
| mac-miR171b-3p.1 | Ma08_t16560.1 | MaNSP2 | 2 | 396 | 416 | UUGAGCCGUGCCAAUAUCACG | GGUGAUAUUGGUUCGGCUCAA | Cleavage |
| mac-miR171b-3p.3 | Ma04_t07170.1 | MaSCL15-3 | 2 | 502 | 521 | UUGAGCCGUGCCAAUAUCAC | GUGAUAUUGUCGCGGCUCAA | Translation |
| mac-miR171b-3p.3 | Ma04_t03560.1 | MaSCL15-2 | 2 | 541 | 560 | UUGAGCCGUGCCAAUAUCAC | GUGAUAUUGUCGCGGCUCAA | Translation |
| mac-miR171b-3p.3 | Ma03_t31190.1 | MaSCL15-1 | 2 | 541 | 560 | UUGAGCCGUGCCAAUAUCAC | GUGAUAUUGUCGCGGCUCAA | Translation |
| mac-miR171b-3p.3 | Ma08_t16560.1 | MaNSP2 | 2 | 397 | 416 | UUGAGCCGUGCCAAUAUCAC | GUGAUAUUGGUUCGGCUCAA | Cleavage |
| mac-miR171b.1 | Ma11_t19440.1 | MaSCL27-4 | 2 | 1314 | 1334 | UGAUUGAGCCGCGUCAAUAUC | GAUAUUGGCACGGCUCAAUCA | Cleavage |
| mac-miR171b.3 | Ma08_t16560.1 | MaNSP2 | 2 | 396 | 416 | UUGAGCCGCGCCAAUAUCACA | GGUGAUAUUGGUUCGGCUCAA | Cleavage |
| mac-miR171c.2 | Ma04_t03560.1 | MaSCL15-2 | 2 | 543 | 563 | AGAUUGAGCCGCGCCAAUAUC | GAUAUUGUCGCGGCUCAAUCA | Cleavage |
| mac-miR171c.2 | Ma04_t07170.1 | MaSCL15-3 | 2 | 504 | 524 | AGAUUGAGCCGCGCCAAUAUC | GAUAUUGUCGCGGCUCAAUCA | Cleavage |
| mac-miR171c.2 | Ma03_t31190.1 | MaSCL15-1 | 2 | 543 | 563 | AGAUUGAGCCGCGCCAAUAUC | GAUAUUGUCGCGGCUCAAUCA | Cleavage |
| mac-miR171d | Ma08_t16560.1 | MaNSP2 | 2 | 397 | 416 | UUGAGCCGCGCCAAUAUCAC | GUGAUAUUGGUUCGGCUCAA | Cleavage |
| mac-miR171f | Ma07_t06770.1 | MaSCL6-2 | 2 | 1122 | 1142 | UAAUUGAGCCGUGCCAAUAUC | GAUAUUGGCGCGGCUCAAUCA | Cleavage |
| mac-miR171f | Ma08_t10950.1 | MaSCL27-2 | 2 | 1299 | 1319 | UAAUUGAGCCGUGCCAAUAUC | GAUAUUGGCGCGGCUCAAUCA | Cleavage |
| mac-miR171f | Ma04_t25650.1 | MaSCL6-1 | 2 | 1146 | 1166 | UAAUUGAGCCGUGCCAAUAUC | GAUAUUGGCGCGGCUCAAUCA | Cleavage |
| mac-miR171f | Ma11_t04080.1 | MaSCL27-3 | 2 | 1311 | 1331 | UAAUUGAGCCGUGCCAAUAUC | GAUAUUGGCGCGGCUCAAUCA | Cleavage |
| mac-miR171f | Ma05_t23270.1 | MaSCL27-1 | 2 | 1296 | 1316 | UAAUUGAGCCGUGCCAAUAUC | GAUAUUGGCGCGGCUCAAUCA | Cleavage |
| mac-miR171f | Ma06_t38750.1 | MaSCL22 | 2 | 426 | 446 | UAAUUGAGCCGUGCCAAUAUC | GAUAUUGGCGCGGCUCAAUCA | Cleavage |
| mac-miR171f.2 | Ma08_t16560.1 | MaNSP2 | 2 | 396 | 416 | UUGAGCCGCGCCAAUAUCACU | GGUGAUAUUGGUUCGGCUCAA | Cleavage |
| mac-miR171h.2 | Ma11_t19440.1 | MaSCL27-4 | 2 | 1314 | 1334 | UGGUUGAGCCGCGCCAAUAUC | GAUAUUGGCACGGCUCAAUCA | Cleavage |
| mac-miR171i-3p | Ma03_t31190.1 | MaSCL15-1 | 2 | 543 | 563 | GGAUUGAGCCGCGUCAAUAUC | GAUAUUGUCGCGGCUCAAUCA | Cleavage |
| mac-miR171i-3p | Ma04_t07170.1 | MaSCL15-3 | 2 | 504 | 524 | GGAUUGAGCCGCGUCAAUAUC | GAUAUUGUCGCGGCUCAAUCA | Cleavage |
| mac-miR171i-3p | Ma04_t03560.1 | MaSCL15-2 | 2 | 543 | 563 | GGAUUGAGCCGCGUCAAUAUC | GAUAUUGUCGCGGCUCAAUCA | Cleavage |
| mac-miR171k | Ma04_t03560.1 | MaSCL15-2 | 2 | 543 | 563 | GGAUUGAGCCGCGCCAAUAUC | GAUAUUGUCGCGGCUCAAUCA | Cleavage |
| mac-miR171k | Ma03_t31190.1 | MaSCL15-1 | 2 | 543 | 563 | GGAUUGAGCCGCGCCAAUAUC | GAUAUUGUCGCGGCUCAAUCA | Cleavage |
| mac-miR171k | Ma04_t07170.1 | MaSCL15-3 | 2 | 504 | 524 | GGAUUGAGCCGCGCCAAUAUC | GAUAUUGUCGCGGCUCAAUCA | Cleavage |
| mac-miR408b | Ma04_t07170.1 | MaSCL15-3 | 2 | 1290 | 1310 | UGCACUGCCUCUUCCCUGGCU | AGCUGGGGAAGGGGCGGUGCA | Cleavage |
| mac-miR408d | Ma04_t07170.1 | MaSCL15-3 | 2 | 1291 | 1310 | UGCACUGCCUCUUCCCUGGC | GCUGGGGAAGGGGCGGUGCA | Cleavage |
| mac-miR171 | Ma11_t19440.1 | MaSCL27-4 | 2.5 | 1311 | 1331 | UUGAGCCGCGUCAAUAUCUCC | UGGGAUAUUGGCACGGCUCAA | Cleavage |
| mac-miR171 | Ma04_t07170.1 | MaSCL15-3 | 2.5 | 501 | 521 | UUGAGCCGCGUCAAUAUCUCC | CGUGAUAUUGUCGCGGCUCAA | Translation |
| mac-miR171 | Ma04_t03560.1 | MaSCL15-2 | 2.5 | 540 | 560 | UUGAGCCGCGUCAAUAUCUCC | CGUGAUAUUGUCGCGGCUCAA | Translation |
| mac-miR171 | Ma03_t31190.1 | MaSCL15-1 | 2.5 | 540 | 560 | UUGAGCCGCGUCAAUAUCUCC | CGUGAUAUUGUCGCGGCUCAA | Translation |
| mac-miR171a.3 | Ma11_t19440.1 | MaSCL27-4 | 2.5 | 1310 | 1330 | UGAGCCGCGCCAAUAUCACAU | AUGGGAUAUUGGCACGGCUCA | Cleavage |
| mac-miR171b-3p.2 | Ma11_t19440.1 | MaSCL27-4 | 2.5 | 1311 | 1331 | UUGAGCCGCGUCAAUAUCUCU | UGGGAUAUUGGCACGGCUCAA | Cleavage |
| mac-miR171b-3p.2 | Ma04_t07170.1 | MaSCL15-3 | 2.5 | 501 | 521 | UUGAGCCGCGUCAAUAUCUCU | CGUGAUAUUGUCGCGGCUCAA | Translation |
| mac-miR171b-3p.2 | Ma03_t31190.1 | MaSCL15-1 | 2.5 | 540 | 560 | UUGAGCCGCGUCAAUAUCUCU | CGUGAUAUUGUCGCGGCUCAA | Translation |
| mac-miR171b-3p.2 | Ma04_t03560.1 | MaSCL15-2 | 2.5 | 540 | 560 | UUGAGCCGCGUCAAUAUCUCU | CGUGAUAUUGUCGCGGCUCAA | Translation |
| mac-miR171b.3 | Ma11_t19440.1 | MaSCL27-4 | 2.5 | 1311 | 1331 | UUGAGCCGCGCCAAUAUCACA | UGGGAUAUUGGCACGGCUCAA | Cleavage |
| mac-miR171c.2 | Ma11_t19440.1 | MaSCL27-4 | 2.5 | 1314 | 1334 | AGAUUGAGCCGCGCCAAUAUC | GAUAUUGGCACGGCUCAAUCA | Cleavage |
| mac-miR171d | Ma11_t19440.1 | MaSCL27-4 | 2.5 | 1312 | 1331 | UUGAGCCGCGCCAAUAUCAC | GGGAUAUUGGCACGGCUCAA | Cleavage |
| mac-miR171f.2 | Ma11_t19440.1 | MaSCL27-4 | 2.5 | 1311 | 1331 | UUGAGCCGCGCCAAUAUCACU | UGGGAUAUUGGCACGGCUCAA | Cleavage |
| mac-miR171k | Ma11_t19440.1 | MaSCL27-4 | 2.5 | 1314 | 1334 | GGAUUGAGCCGCGCCAAUAUC | GAUAUUGGCACGGCUCAAUCA | Cleavage |
| mac-miR171m | Ma11_t19440.1 | MaSCL27-4 | 2.5 | 1311 | 1331 | UUGAGCCGCGUCAAUAUCUCA | UGGGAUAUUGGCACGGCUCAA | Cleavage |
| mac-miR171m | Ma04_t07170.1 | MaSCL15-3 | 2.5 | 501 | 521 | UUGAGCCGCGUCAAUAUCUCA | CGUGAUAUUGUCGCGGCUCAA | Translation |
| mac-miR171m | Ma04_t03560.1 | MaSCL15-2 | 2.5 | 540 | 560 | UUGAGCCGCGUCAAUAUCUCA | CGUGAUAUUGUCGCGGCUCAA | Translation |
| mac-miR171m | Ma03_t31190.1 | MaSCL15-1 | 2.5 | 540 | 560 | UUGAGCCGCGUCAAUAUCUCA | CGUGAUAUUGUCGCGGCUCAA | Translation |
| mac-miR171n | Ma11_t19440.1 | MaSCL27-4 | 2.5 | 1311 | 1331 | UUGAGCCGCGUCAAUAUCUUA | UGGGAUAUUGGCACGGCUCAA | Cleavage |
| mac-miR171n | Ma04_t07170.1 | MaSCL15-3 | 2.5 | 501 | 521 | UUGAGCCGCGUCAAUAUCUUA | CGUGAUAUUGUCGCGGCUCAA | Translation |
| mac-miR171n | Ma04_t03560.1 | MaSCL15-2 | 2.5 | 540 | 560 | UUGAGCCGCGUCAAUAUCUUA | CGUGAUAUUGUCGCGGCUCAA | Translation |
| mac-miR171n | Ma03_t31190.1 | MaSCL15-1 | 2.5 | 540 | 560 | UUGAGCCGCGUCAAUAUCUUA | CGUGAUAUUGUCGCGGCUCAA | Translation |
| mac-miR5658 | Ma11_t18500.1 | MaSCR-3 | 2.5 | 101 | 121 | AUGAUGAUGAUGAUGAUGAAA | CCACAUCAUCAUCGUCAUCAC | Cleavage |
| mac-miR171f | Ma03_t31190.1 | MaSCL15-1 | 3 | 543 | 563 | UAAUUGAGCCGUGCCAAUAUC | GAUAUUGUCGCGGCUCAAUCA | Cleavage |
| mac-miR171f | Ma04_t03560.1 | MaSCL15-2 | 3 | 543 | 563 | UAAUUGAGCCGUGCCAAUAUC | GAUAUUGUCGCGGCUCAAUCA | Cleavage |
| mac-miR171f | Ma04_t07170.1 | MaSCL15-3 | 3 | 504 | 524 | UAAUUGAGCCGUGCCAAUAUC | GAUAUUGUCGCGGCUCAAUCA | Cleavage |
| mac-miR171i-3p | Ma11_t19440.1 | MaSCL27-4 | 3 | 1314 | 1334 | GGAUUGAGCCGCGUCAAUAUC | GAUAUUGGCACGGCUCAAUCA | Cleavage |
| mac-miR172a.1 | Ma01_t22010.1 | MaCIGR2-1 | 3 | 269 | 288 | AGAAUCUUGAUGAUGCUGCAU | CCGCAUC-UCAUCAAGAUUCU | Cleavage |
| mac-miR172c-3p | Ma01_t22010.1 | MaCIGR2-1 | 3 | 271 | 288 | AGAAUCUUGAUGAUGCUGC | GCAUC-UCAUCAAGAUUCU | Cleavage |
| mac-miR172c.1 | Ma01_t22010.1 | MaCIGR2-1 | 3 | 269 | 288 | AGAAUCUUGAUGAUGCUGCAG | CCGCAUC-UCAUCAAGAUUCU | Cleavage |
| mac-miR396a.1 | Ma07_t06770.1 | MaSCL6-2 | 3 | 182 | 202 | UCCACAGGCUUUCUUGAACAU | CGGUUCUGGAAAGCCUGCGGA | Cleavage |
| mac-miR396e-5p | Ma07_t06770.1 | MaSCL6-2 | 3 | 182 | 202 | UCCACAGGCUUUCUUGAACUG | CGGUUCUGGAAAGCCUGCGGA | Cleavage |
| mac-miR396g | Ma07_t06770.1 | MaSCL6-2 | 3 | 182 | 202 | UCCACAGGCUUUCUUGAACGG | CGGUUCUGGAAAGCCUGCGGA | Cleavage |
| mac-miR408-3p.1 | Ma04_t07170.1 | MaSCL15-3 | 3 | 1291 | 1310 | UGCACUGCCUCUUCCCUGCC | GCUGGGGAAGGGGCGGUGCA | Cleavage |
| mac-miR408-3p.2 | Ma04_t07170.1 | MaSCL15-3 | 3 | 1291 | 1311 | AUGCACUGCCUCUUCCCUGGC | GCUGGGGAAGGGGCGGUGCAA | Cleavage |
| mac-miR408-3p.3 | Ma04_t07170.1 | MaSCL15-3 | 3 | 1291 | 1311 | CUGCACUGCCUCUUCCCUGGC | GCUGGGGAAGGGGCGGUGCAA | Cleavage |
| mac-miR5234 | Ma04_t26390.1 | MaSCL28-3 | 3 | 102 | 122 | UUUUGUUGUGGAUGGCAGAAG | GCAUUGCCAUCCGCAGCAGAA | Cleavage |
